# Performance Analysis of an Integrated Multi-Stage System for Coffee Industry Wastewater Treatment

**DOI:** 10.3390/ma19102098

**Published:** 2026-05-16

**Authors:** Angelika Skorupa, Małgorzata Worwąg, Mariusz Kowalczyk, Paulina Szuniewicz

**Affiliations:** Faculty of Infrastructure and Environmental, Czestochowa University of Technology, 42-200 Czestochowa, Poland; malgorzata.worwag@pcz.pl (M.W.); mariusz.kowalczyk@pcz.pl (M.K.); s134815ps@student.pcz.pl (P.S.)

**Keywords:** coffee, biological treatment, activated carbon, membrane filtration, disinfection, wastewater treatment

## Abstract

**Highlights:**

- Integrated treatment for coffee wastewater was successfully developed.
- Combined SBR, activated carbon, and membrane filtration ensured high removal rates.
- Dual disinfection with ozone and UV improved final water quality.
- Achieved high organic and nutrient removal efficiencies.
- Demonstrated potential for wastewater reuse in industrial applications.

**Abstract:**

Wastewater generated during the processing of roasted coffee, including instant coffee, remains relatively unknown in the literature. However, it is characterized by a high organic load and the presence of caffeine, phenolic compounds, and melanoidins. Its properties pose significant environmental and technological challenges, limiting the effectiveness of conventional treatment methods. The research aimed to evaluate the effectiveness of an integrated, multi-stage wastewater treatment system that reflects the process of roasted coffee extraction. The developed technological sequence included biological treatment, activated carbon sorption, membrane filtration, and disinfection using ozone and UV radiation. The experiments used synthetic wastewater containing an extract of roasted coffee beans to simulate the contaminants typically found in instant coffee production and the cleaning of processing equipment. The integrated treatment system enabled the removal of total organic carbon (82.4–95.4%), ammonium nitrogen (0–77.4%), and phosphates (0–39.9%), and a reduction in turbidity of 96.3–99.8% at pH 4.02–7.25. The results confirm the system’s high efficiency and its potential for treating complex coffee wastewater, while also highlighting the need for further research into the selection of more favorable process parameters.

## 1. Introduction

The coffee industry is a key sector of the global economy. Coffee is the second most valuable export commodity, second only to crude oil. It plays an important role in both producing and consuming countries [1]. In recent years, the coffee market has been growing rapidly. According to data from the International Coffee Organization (ICO), global production in the 2022–2023 season amounted to 168.2 million 60-kg bags of coffee, while in the 2023–2024 coffee season, this figure rose to 178 million bags. At the same time, global coffee consumption increased from 173.1 million to 177 million 60-kg bags over these years [2].

There are three basic methods of processing coffee beans: dry, semi-dry, and wet. Of these technologies, the wet method is the most water-intensive. This is due to the process’s complexity, which includes stages such as peeling, washing, and fermentation. As a result, significant amounts of wastewater with a high organic pollutant load are generated already at the initial stage of raw material processing. It should be emphasized that coffee production is a multi-stage process, starting with harvesting and processing the beans, followed by drying, storage, roasting, grinding, and, in the case of instant coffee, extraction, concentration, and drying. Each of these stages can generate wastewater with different physicochemical properties and varying qualitative and quantitative composition [1,3,4].

Wastewater generated during coffee bean processing poses a significant environmental and technological challenge due to its volume and complex chemical composition [5]. It is characterized by a low pH (3.5–4.5) due to organic acids produced during the fermentation and decomposition of plant matter. Discharging such wastewater into the environment without prior treatment can have serious ecological consequences, including acidification of aquatic ecosystems and disruption of their biological balance [6,7].

In addition, this wastewater contains high concentrations of organic matter, including tannins, polyphenols, alkaloids (e.g., caffeine), sugars, and proteins. The presence of these compounds results in elevated Chemical Oxygen Demand (COD) and Biochemical Oxygen Demand (BOD_5_) values and increased wastewater viscosity, which can hinder their natural biodegradation and limit the effectiveness of self-purification processes in the environment [8,9]. In addition, elevated nutrient concentrations, such as nitrogen and phosphorus, can contribute to eutrophication of water bodies [7]. This process leads to the intensive growth of phytoplankton and aquatic vegetation, which, in turn, results in deterioration of water quality, dissolved oxygen deficiency, and conditions conducive to the formation of toxic metabolites and the gradual overgrowth of reservoirs [6,10].

The complex physicochemical nature of this wastewater, including low pH, high concentrations of readily and non-readily biodegradable organic matter, and elevated nutrient levels, means that its uncontrolled discharge poses a substantial threat to both surface and groundwater. Therefore, proper treatment of wastewater generated during coffee bean processing is crucial for minimizing its harmful impact on the environment.

However, the coffee industry does not end with plantations and processing plants—cafes and catering establishments, which prepare drinks for thousands of customers every day, are also a significant source of waste. In Poland, the number of catering establishments increased from 83,937 in 2022 to 93,306 in 2023, representing an 11.2% increase over the previous year [11]. Although not all of these establishments are cafés, their number is growing; with this growth, the production of coffee waste is also rising.

The data shows that one cup of coffee generates an average of 8–10 g of coffee grounds. Assuming each of the nearly 100,000 catering establishments in Poland serves an average of 100 cups of coffee per day, the country’s annual coffee ground production may amount to approximately 80,000 tons. This is a huge amount of waste, most of which ends up in landfills or incinerators [12].

In addition to coffee grounds and wastewater, other waste products are generated, including coffee pulp and coffee husks. For example, processing 6250 kg of ripe coffee cherries requires 125,000 L of water and yields 1000 kg of green coffee [13]. At the same time, this generates up to 25,000 L of wastewater and 2500 tons of pulp. Improper disposal of such a significant amount of waste can contribute to river pollution, rendering them unusable for domestic, recreational, and economic purposes [14]. Wastewater from coffee bean processing can also degrade sewage infrastructure and wastewater treatment plants, thereby increasing operating costs. Although selected waste has the potential to be reused, for example, in the production of compost or biofuels, its improper management poses a threat to the environment [6,15,16].

Despite the growing importance of the coffee industry globally, scientific literature still lacks sufficiently comprehensive information on the properties of wastewater generated at all stages of coffee production and processing. Previous studies have primarily focused on wastewater from the wet processing of beans, while other stages, such as roasting or instant coffee production, are analyzed much less frequently. This gap requires further in-depth analysis, as wastewater—owing to its high organic matter load, the presence of poorly biodegradable compounds, and compositional variability—poses significant environmental and technological challenges. Expanding knowledge in this area, based on an analysis of available literature data and our own research, seems crucial for the development of effective and sustainable methods for its treatment.

**Figure 1:**
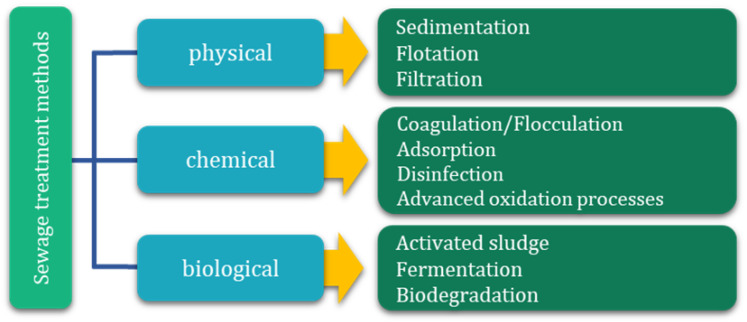
Examples of wastewater treatment methods [15,19,20,21,22].

The dominant method of water and wastewater management worldwide remains the centralized sewerage system, which collects domestic, commercial, and industrial wastewater. Approximately 60% of the global population is connected to sewerage networks, but not all collected wastewater is effectively treated before discharge into the environment. In the context of growing coffee production and increasing industrial wastewater generation, the management of this wastewater becomes particularly important. In the case of wastewater generated during coffee bean processing, many companies limit themselves to preliminary treatment before discharging it into the municipal sewage system. Although this procedure meets the minimum formal requirements, it does not consistently ensure the effective removal of high loads of organic matter, phenolic compounds, or caffeine. Traditional sewage systems and conventional wastewater treatment plants, even in developed urban areas, are not always equipped to remove specific, difficult-to-biodegrade compounds characteristic of coffee-industry wastewater effectively. As a result, some of these pollutants may enter water bodies and the soil environment. Therefore, there is a need to develop more effective, integrated strategies for treating wastewater generated at various stages of coffee bean processing to minimize environmental impacts and reduce the risk of secondary pollution of ecosystems [17,18]. Wastewater treatment processes can be classified as physical, chemical, and biological (Figure 1), with increasing emphasis on the integration of these methods in multi-stage technological systems.

The use of a technological process involving biological treatment, sorption on filter media (e.g., commonly used activated carbon), membrane filtration, and disinfection (e.g., ozonation or UV radiation) shows significant potential for wastewater with high organic load and recalcitrant compounds. This approach can not only enable compliance with applicable environmental standards but also facilitate the recovery of water that meets parameters suitable for reuse, for example, for industrial or agricultural purposes [4,14,21,23].

Despite growing interest in coffee-industry wastewater management, there remains insufficient data in the literature on comprehensive, integrated treatment systems that account for the diverse nature of wastewater generated at different stages of production. This underscores the need for further interdisciplinary research to develop effective technologies that mitigate the environmental pressures of this rapidly evolving sector.

This study aimed to evaluate the effectiveness of an integrated treatment system comprising biological processes, activated carbon sorption, membrane filtration, and two-stage disinfection (ozone/UV) for the removal of difficult organic pollutants.

The novelty of the study lies not in the individual technologies, which are well established, but in their integration and application to a relatively underexplored type of wastewater, as well as in the assessment of their combined performance.

Synthetic wastewater supplemented with roasted coffee bean extract was used to approximate effluents generated during roasted coffee extraction and cleaning processes in instant coffee production. This type of wastewater contains high levels of organic matter, which may exhibit limited biodegradability. In contrast to most studies focusing on green coffee processing, this work addresses a different pollutant profile.

A simplified and reproducible model wastewater enabled preliminary, controlled investigations aimed at identifying favorable operating conditions for individual stages of the treatment system. The results are indicative and provide a basis for further research using real industrial wastewater.

The study assessed both the effectiveness of individual processes and potential interactions within a multi-stage system (biological–sorption–membrane–advanced disinfection), for which data remain limited. The results suggest that process integration may enhance removal efficiency and improve final effluent quality.

Overall, the study provides preliminary insights into the integrated treatment of wastewater containing roasted coffee extraction products. Its contribution lies in the evaluation of a multi-stage system under controlled conditions, the use of a representative model of wastewater, and the identification of potential process interactions relevant to future optimization of treatment strategies.

## 2. Materials and Methods

**Figure 2:**
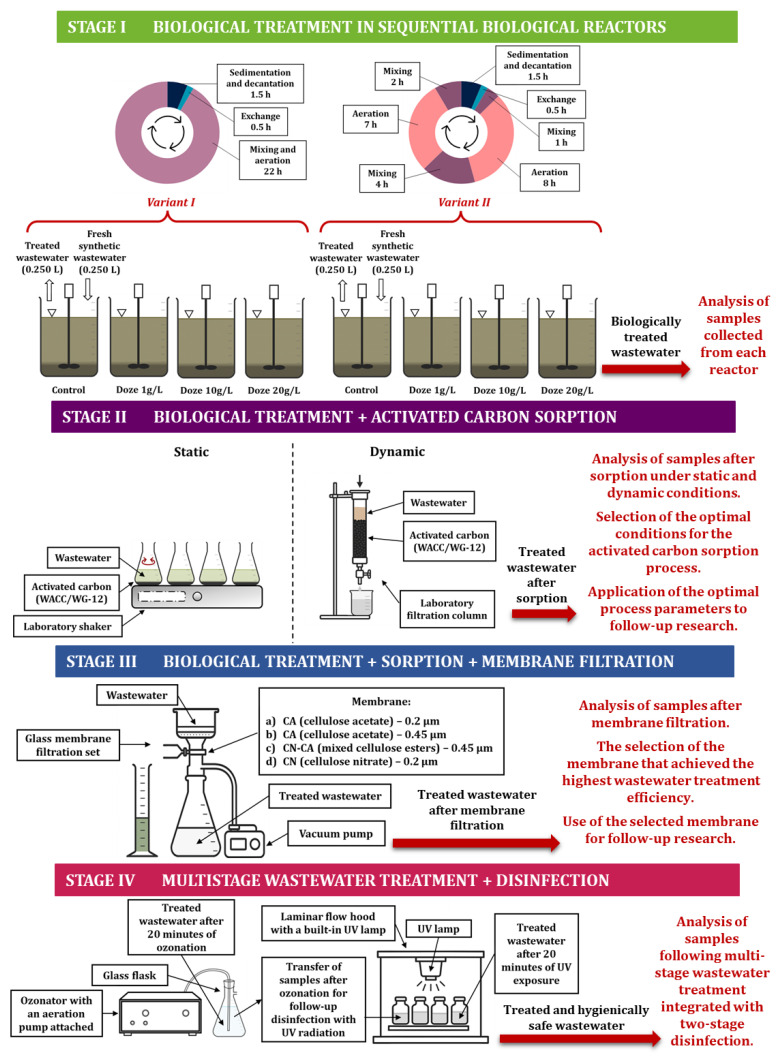
General scheme of the research being conducted (own elaboration).

The research used a multi-stage, integrated technological process for wastewater treatment, schematically shown in Figure 2. This system included, in sequence, biological treatment, sorption, membrane filtration, and final disinfection, creating a coherent wastewater treatment system with a gradually increasing level of technological advancement.

The methods used were deliberately selected—they are widely described in the scientific literature and commonly used in engineering practice at both laboratory and technical scales. Their effectiveness in removing organic, biogenic, and microbiological pollutants has been repeatedly confirmed in experimental studies and industrial implementations.

The selection of well-known, well-characterized technological processes enabled not only the assessment of their effectiveness relative to the type of wastewater under study but also the analysis of their potential integration into a single technological line. This approach increases the application potential.

### 2.1. Research Substrate

The research substrate was synthetic wastewater prepared in accordance with the PN-72/C-04550/09 standard [24], with the addition of a model contaminant, a coffee bean extract. The extract was prepared according to the procedure described by Shanmugam and Gummadi [25], using a 60:40 mixture of Arabica and Robusta coffee beans. To simulate the various pollution levels characteristic of wastewater from the coffee industry, three extract concentrations were used: 1, 10, and 20 g/L. Table 1 presents the general characteristics of the freshly prepared synthetic wastewater. The results shown are the average of three replicates.

The use of synthetic wastewater containing a model contaminant was a deliberate and methodologically justified decision. The main advantage of this approach is the ability to fully control the physicochemical composition of the wastewater, including the concentrations of organic compounds and biogenic components. This made it possible to conduct research under repeatable conditions and to directly compare results between individual experimental series. This approach also facilitates the interpretation of results and the assessment of the impact of specific factors on the effectiveness of treatment processes.

Actual wastewater from coffee processing is characterized by high compositional variability. This is due, among other things, to the type of beans used (Arabica or Robusta), their origin, the processing method employed (dry, wet, or semi-dry), the degree of roasting, and the seasonality of production. Additionally, these effluents may contain variable amounts of phenolic compounds, caffeine, and other substances that can inhibit biological processes. Such high variability in composition makes it difficult to conduct research under controlled conditions and to unambiguously interpret the results obtained.

The use of coffee bean extract as a model contaminant made it possible to replicate the key characteristics of coffee wastewater, such as its high organic content and the presence of aromatic compounds. The use of three different extract concentrations (1, 10, and 20 g/L) in the studies enabled an analysis of the impact of increasing pollutant loads on treatment efficiency and an assessment of the system’s operational stability under various conditions. This made it possible to simulate both typical and elevated pollutant concentrations that may occur in industrial practice.

It should be noted, however, that the use of synthetic wastewater also has its limitations. It does not fully reflect the complex composition of actual wastewater, which contains various components, including suspended solids, colloidal substances, and natural microflora. Furthermore, the absence of certain specific pollutants may affect the efficiency of the treatment processes.

Despite these limitations, the approach adopted is appropriate for the proof-of-concept phase, the aim of which was to evaluate the performance of the proposed technological system under controlled conditions.

### 2.2. Biological Treatment

In the first stage of the research, wastewater was subjected to biological treatment using the activated-sludge process in Sequential Batch Reactors (SBRs). The research used eight laboratory reactor models in the form of cylindrical plastic tanks, each with a working capacity of 2.5 L. Aeration was carried out using an aeration pump with a capacity of 420 L/h, and mechanical stirrers maintained a constant speed of 100 rpm.

The activated sludge used in the research was obtained from the sludge stabilization chamber of a municipal wastewater treatment plant equipped with a SUPERBOS system based on zone-activated sludge technology. Before the actual research stage began, the sludge was adapted to laboratory conditions, a process that continued until the reactors were operating stably.

**Figure 3:**
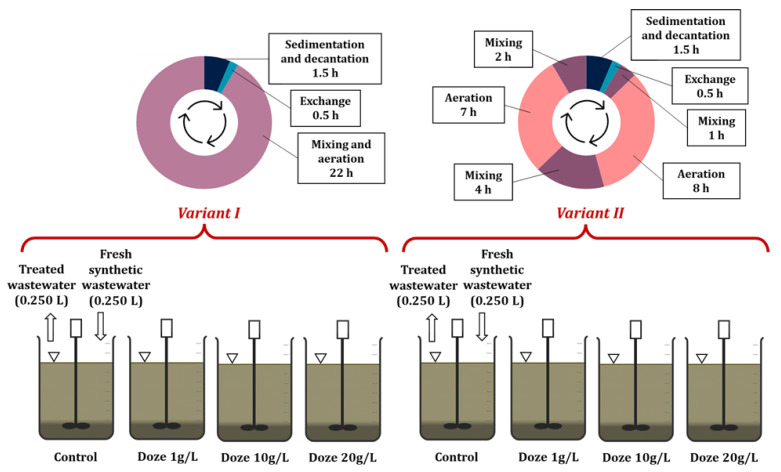
Schematic diagram of a research station for biological wastewater treatment (own elaboration).

Once stable operating conditions were established in the reactors, synthetic wastewater containing coffee bean extract at various concentrations began to be fed into selected reactors. The dosing scheme for the synthetic wastewater containing the model contaminant is shown in Figure 3.

Based on the results of preliminary research [23], a Hydraulic Retention Time (HRT) of 10 days was adopted. It was shown that among the analyzed HRT variants (10, 15, and 25 days), the most favorable parameters of biologically treated wastewater were obtained at an HRT of 10 days. For the selected HRT, the daily volume of effluent discharged from each reactor after sedimentation was 0.250 L. After the biologically treated effluent was discharged, the same volume was replenished with freshly prepared synthetic wastewater, both with and without the addition of coffee bean extract (control).

All reactors operated on a 24-h cycle. Two operating variants were used (cycle variant I and cycle variant II). Four reactors operated according to the first variant, and the remaining four according to the second (Figure 3).

In the first variant, the reactor contents were aerated and mixed for 22 h, followed by deactivation of aeration and mixing and sedimentation lasting 1.5 h. Then, the working volume was replaced, and the next cycle began. In the second variant, alternating aeration and mixing phases were carried out for 22 h, followed by 1.5 h of sedimentation and replacement of the working volume, as in the first variant. Detailed parameters of the reactor operating cycles are presented in Table 2.

With HRT set to 10 days, the daily wastewater volume discharged after sedimentation was calculated as 0.250 L per reactor. A simplified diagram of the research setup is shown in Figure 3.

The actual research was preceded by a stage of adapting activated sludge microorganisms to synthetic wastewater without the addition of coffee bean extract. Once the reactors had stabilized, synthetic wastewater containing coffee bean extract was dosed at concentrations of 1, 10, and 20 g/L, in accordance with the diagram shown in Figure 3.

After 10 days of running the process with the addition of coffee bean extract, analyses were performed to determine: pH, turbidity, total organic carbon (TOC), ammonium nitrogen, and phosphates.

### 2.3. Sorption on Activated Carbon

The biologically treated wastewater was then subjected to an additional sorption treatment stage. Activated carbon, commonly used in water and wastewater treatment due to its high specific surface area and developed porous structure conducive to the adsorption of organic and inorganic compounds, was used as the sorbent. The research compared the effectiveness of two commercial carbon adsorbents: WG-12 (manufactured by Gryfskand, Gryfino, Poland) and WACC (manufactured by Elbar—Katowice, Ltd., Katowice, Poland).

A description of the activated carbons used in the research is presented in Table 3.

**Figure 4:**
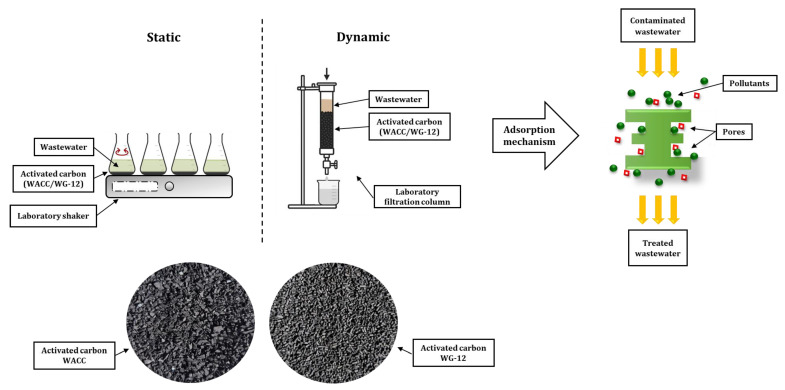
Schematic diagram illustrating the activated carbon sorption process (own elaboration).

The sorption process was carried out in two technological variants: static (batch) and dynamic (flow). This approach made it possible to assess the effectiveness of pollutant removal under equilibrium conditions and in a system resembling the actual technological solutions used in engineering practice. A diagram of the sorption process mechanism is shown in Figure 4.

In the static variant, 1.0 g of activated carbon was added to conical flasks, which were then filled with 0.125 L of biologically treated wastewater from the previous stage of the research. The samples prepared in this way were placed on a laboratory shaker and mixed for 2 h at a frequency of 140 c.p.m. The purpose of intensive shaking was to ensure adequate contact between the liquid phase and the surface of the adsorbent. After shaking, the samples were left to rest, allowing further sorption processes to take place and equilibrium to be reached. Analyses were performed 24, 48, and 72 h after the start of the experiment, which allowed for the assessment of the dynamics of changes in the tested parameters over time and the stability of the obtained purification effects.

At the same time, the sorption process was carried out in a dynamic system using a filter column filled with activated carbon. The height of the sorption layer was 20 cm, while the diameter of the column was 3 cm^3^. Biologically treated wastewater in a volume of 125 mL was passed through a stationary adsorbent bed. The average linear velocity of the solution flow through the sorption layer was estimated at 0.2 cm/min. During the flow through the column, the dissolved compounds were adsorbed on the surface and in the pores of the activated carbon. The dynamic system enabled the evaluation of the process efficiency under flow conditions characteristic of sorption columns used in technological installations.

The samples obtained after the sorption process—both in the static and dynamic variants—were analyzed for pH, turbidity, phosphate content, and total organic carbon (TOC). These parameters were selected as indicators of pollutant removal and wastewater quality improvement after the post-treatment stage.

The research allowed for a comparison of the effectiveness of two types of activated carbon (WACC and WG-12) depending on the process method and an assessment of the potential of sorption as a stage of wastewater post-treatment after biological treatment in SBRs.

### 2.4. Membrane Filtration

After completion of the sorption process, the obtained samples were subjected to a further purification stage—membrane filtration under pressure. Samples obtained from sorption carried out under dynamic conditions were selected for further research. This selection was justified both by technological considerations and by the results obtained earlier.

The research compared the effectiveness of four types of membranes differing in both material and pore diameter:

1. CA (cellulose acetate)—0.2 µm (manufactured by Ahlstrom-Munksjö Germany GmbH, Bärenstein, Germany),
2. CA (cellulose acetate)—0.45 µm (manufactured by Ahlstrom-Munksjö Germany GmbH, Bärenstein, Germany),
3. CN-CA (mixed cellulose esters)—0.45 µm (manufactured by Ahlstrom-Munksjö Germany GmbH, Bärenstein, Germany),
4. CN (cellulose nitrate)—0.2 µm (manufactured by Sartorius, Germany).

Four membranes were used, differing in both material and pore size, which directly affected their filtration properties. The membranes used had a diameter of 47 mm.

Membranes with 0.2 µm pores (CA; CN) exhibited similar flow rates for water of approximately 24–25 mL/min/cm^2^/bar, but differed significantly in their bubble point. The CN membrane exhibited a significantly higher value (4.2 bar) compared to the CA membrane (2.9 bar), indicating a more uniform pore structure and higher filtration efficiency. The CN 0.2 µm membrane is hydrophilic and has a thickness of 130 µm, whereas the CA 0.2 µm membrane is also hydrophilic with a thickness of 120 µm.

Membranes with a larger pore diameter (0.45 µm) exhibit a significantly higher flow rate for water. The CA membrane achieves approximately 65 mL/min/cm^2^/bar at a bubble point of 2.4 bar, while the CN-CA membrane exhibits a similar, slightly more varied range of flow rates for water (approx. 60–70 mL/min/cm^2^/bar) and a bubble point of 2.1–2.4 bar. Both the CA 0.45 µm membrane and the CN-CA 0.45 µm membrane are hydrophilic. The CA 0.45 µm membrane has a thickness of 120 µm, whereas the CN-CA 0.45 µm membrane has a thickness in the range of 120–150 µm.

The use of membranes with two pore sizes (0.2 and 0.45 µm) allowed for the assessment of the impact of separation accuracy on permeate quality, while the use of different polymer materials enabled the analysis of the impact of the physicochemical properties of membranes on the efficiency of the process.

**Figure 5:**
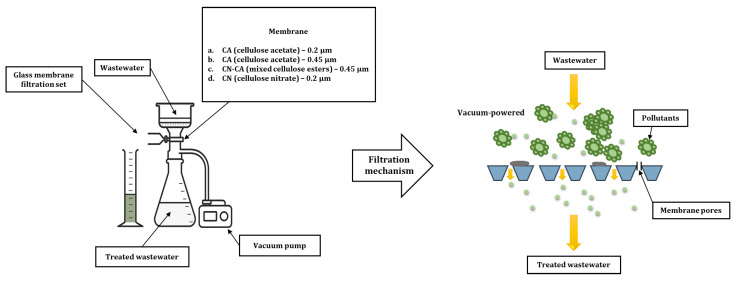
Schematic diagram of the membrane filtration process (own elaboration).

Filtration was carried out using a glass filtration set connected to a pressure pump. The average operating pressure during the process was 0.08 MPa, which ensured stable flow through the membrane and repeatability of the experimental conditions. A diagram of the membrane filtration mechanism is shown in Figure 5.

The pH value and turbidity were determined in the samples obtained after filtration as basic indicators of the process’s efficiency. The analysis of these parameters enabled the comparison of the effectiveness of individual membranes and the assessment of the validity of using an integrated system, dynamic sorption–membrane filtration, as an effective solution for the additional treatment of wastewater after the biological process.

### 2.5. Disinfection with Ozone and UV Radiation

The final stage of wastewater treatment was two-stage disinfection: ozonation followed by exposure to UV radiation. The use of an integrated O_3_/UV system was intended not only to inactivate microorganisms but also to further oxidize any remaining organic compounds in the wastewater after the previous treatment stages (biological, sorption, and membrane).

In the first stage, the samples were exposed to ozone for 20 min. The ozonation process was carried out using the IMPOZ MINI 2 ozonator, a portable ozone generator manufactured by OPTEL Ltd. (Wrocław, Poland), designed to generate a mixture of ozone and air with a maximum concentration of 10 g O_3_/m^3^.

The ozonation of treated wastewater samples was performed at a constant flow rate of ozonated air of 10 L/h. Ozone, as a strong oxidant, leads to the inactivation of microorganisms. At the same time, it can cause partial mineralization or transformation of selected organic compounds into forms more susceptible to further oxidation processes.

In the second stage, the samples were irradiated with UV-C radiation with a wavelength of 253.7 nm, emitted by a bacteriological lamp, for 20 min. The disinfection process was carried out using a UV-C lamp installed in a laminar flow cabinet, with a typical power of approximately 30 W. The intensity of UV radiation in the laminar flow hood is very high, as it is designed to ensure effective sterilization. The use of UV radiation after ozonation increased the effectiveness of the disinfection process and reduced the risk of secondary microflora growth.

The exposure times to both ozone and UV radiation were selected based on the results of preliminary research [23], which analyzed the effectiveness of different exposure time variants of the process. The adopted parameters were considered optimal in terms of disinfection efficiency and energy efficiency of the process.

After each stage of wastewater treatment, the samples were subjected to the following analyses:

1. pH by the electrometric method using a pH meter with an electrode in accordance with the methodology described by Hermanowicz et al. (1976) [28];
2. Phosphates by the spectrophotometric method with ascorbic acid in accordance with the methodology described by Hermanowicz et al. (1976) [28];
3. Total organic carbon (TOC) was determined in accordance with PN-EN 1484 [29] using a Multi N/C 3100 TOC analyzer from Analytik Jena (manufactured in Jena, Germany);
4. Ammoniacal nitrogen was determined spectrophotometrically using ready-made tests from HACH (method 8155 Powder Pillows—salicylate method);
5. Turbidity was read from a HACH laboratory turbidimeter.

Each analysis was performed in triplicate to ensure the reliability and reproducibility of the results obtained. The values obtained showed very high consistency across the individual replicates, indicating the high precision of the analytical method used. For this reason, the results section presents average values that representatively reflect the obtained results without losing significant information resulting from the small data spread.

## 3. Results

### 3.1. Biological Treatment

After 10 days of conducting the process with the addition of coffee bean extract, analyses were performed to determine: pH, turbidity, total organic carbon (TOC), ammonium nitrogen, and phosphates.

**Figure 6:**
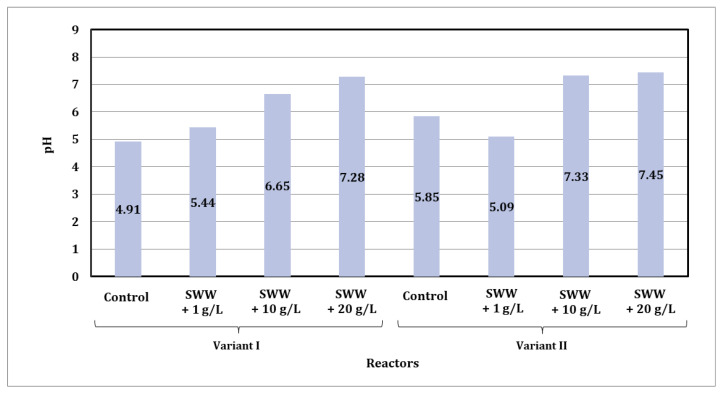
pH values obtained after biological treatment of synthetic wastewater (SWW) and synthetic wastewater with coffee extract (own elaboration).

Based on the test results, it was found that the pH of biologically treated wastewater ranged from acidic and slightly acidic to neutral (Figure 6). Wastewater containing a higher amount of coffee extract had a significantly higher pH value. In samples from reactors in which synthetic wastewater was treated without the addition of coffee extract, the pH value ranged from 4.91 to 5.85. For samples from reactors where coffee extract was added to synthetic wastewater at a concentration of 1 g/L, the pH value ranged from 5.09 to 5.44, while at a concentration of 10 g/L the range increased to 6.65–7.33. In turn, in samples from reactors where coffee extract was added at a concentration of 20 g/L, the pH value ranged from 7.28 to 7.45.

It was also noted that the use of different phase modules in the reactors affected the pH values of the treated wastewater. Slightly higher pH values were observed in phase II, where alternating aeration and mixing were used.

**Figure 7:**
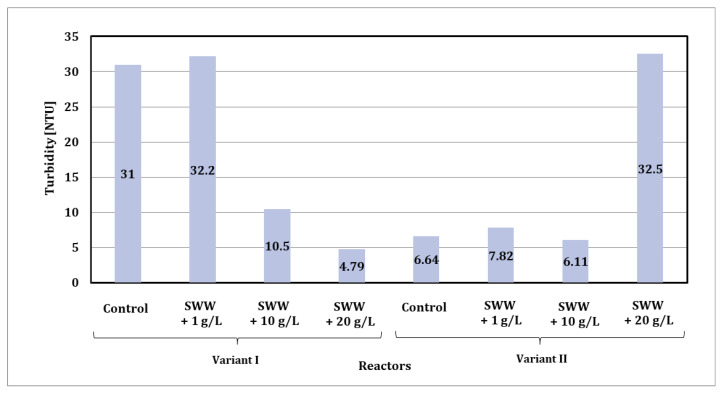
Turbidity values obtained after biological treatment of synthetic wastewater (SWW) and synthetic wastewater with coffee extract (own elaboration).

Based on the results of turbidity tests, significant differences were observed depending on the reactor phase module used and the composition of the wastewater fed into the reactors (Figure 7).

In the case of phase module I, synthetic wastewater without the addition of coffee extract showed a turbidity level of 31.00 NTU. The addition of coffee extract at a concentration of 10 g/L resulted in a significant reduction in turbidity to 10.50 NTU, while at a concentration of 20 g/L, turbidity decreased even further, reaching a value of 4.79 NTU. It is worth noting that the sample with the lowest extract concentration (1 g/L) had higher turbidity (32.2 NTU), similar to the control value.

In phase II, lower turbidity values were generally observed. In the control sample (synthetic wastewater without the addition of coffee extract), the turbidity was 6.64 NTU. The addition of coffee extract at a concentration of 10 g/L slightly reduced turbidity to 6.10 NTU, and at a concentration of 20 g/L, it reached its lowest value of 5.30 NTU. As in the case of phase I, the lowest concentration of extract (1 g/L) resulted in the highest turbidity among the samples containing extract, amounting to 32.5 NTU.

In summary, the addition of coffee extract to synthetic wastewater introduced into the reactors significantly reduced the turbidity of biologically treated wastewater in both phase modules, especially at higher extract concentrations. The lower turbidity values obtained in the phase II module may be related to a more effective wastewater treatment process resulting from the alternating aeration and mixing system.

**Figure 8:**
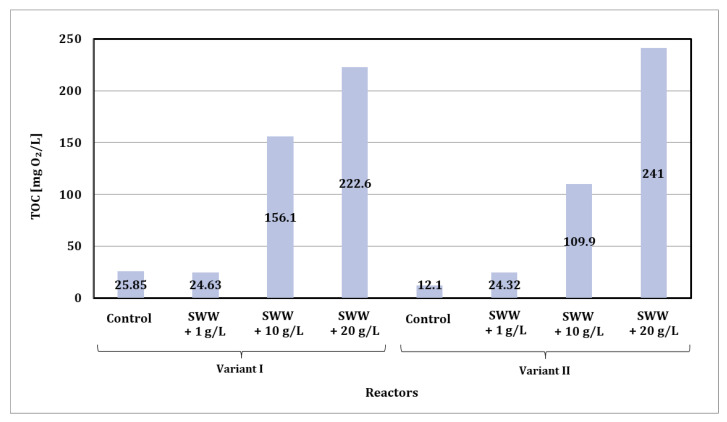
Total organic carbon values obtained after biological treatment of synthetic wastewater (SWW) and synthetic wastewater with coffee extract (own elaboration).

Based on the obtained test results, it was found that the TOC content in biologically treated wastewater depended both on the concentration of coffee extract added to synthetic wastewater and on the phase module used in the biological reactor (Figure 8).

In the case of phase module I, the TOC content in the control samples (synthetic wastewater without the addition of coffee extract) was 25.85 mg O_2_/L. For samples containing coffee extract at a concentration of 1 g/L, this value was slightly lower, at 24.63 mg O_2_/L. At higher concentrations of coffee extract, a significant increase in TOC content was observed: at a concentration of 10 g/L, the content was 156.1 mg O_2_/L, and at a concentration of 20 g/L, it increased to 222.6 mg O_2_/L.

In phase II, the TOC values in the control samples were lower than in phase I and amounted to 12.10 mg O_2_/L. In samples with the addition of extract at a concentration of 1 g/L, the TOC value increased to 24.32 mg O_2_/L, which is similar to the results from phase I. At higher concentrations of coffee extract, the TOC values were also significantly increased: at a concentration of 10 g/L, they were 109.9 mg O_2_/L, and at a concentration of 20 g/L, they reached their highest value of 241.0 mg O_2_/L.

It was observed that higher doses of coffee extract significantly increased the total organic carbon content in treated wastewater, with the phase II module, characterized by an alternating aeration and mixing system, allowing for a more effective reduction in TOC content in control samples and those with lower extract concentrations compared to the phase I module. In the case of samples with higher extract doses, the differences between the modules were less pronounced.

**Figure 9:**
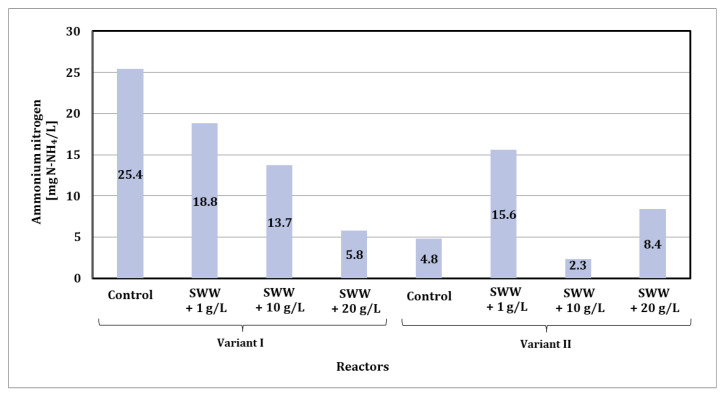
Ammonium nitrogen values obtained after biological treatment of synthetic wastewater (SWW) and synthetic wastewater with coffee extract (own elaboration).

Before the biological treatment process, the concentration of ammonium nitrogen in synthetic wastewater was 6.0 mg N-NH_4_/L. In the case of synthetic wastewater with the addition of coffee extract at concentrations of 1 g/L, 10 g/L, and 20 g/L, these values were 5.50 mg/L, 6.60 mg/L, and 9.30 mg/L, respectively. The biological treatment process significantly reduced these values depending on the reactor phase module used and the amount of coffee extract added (Figure 9).

In the case of phase module I, a clear reduction in ammonium nitrogen concentration was observed with an increase in coffee extract concentration in synthetic wastewater. In control samples without the addition of coffee extract, the ammonium nitrogen concentration was 25.4 mg N-NH_4_/L. With the addition of coffee extract at a concentration of 1 g/L, the ammonium nitrogen level dropped to 18.8 mg N-NH_4_/L, while at a concentration of 10 g/L it decreased to 13.7 mg N-NH_4_/L. The lowest concentration, 5.8 mg N-NH_4_/L, was recorded in samples with the addition of coffee extract at a concentration of 20 g/L.

Different results were obtained for phase II modules. The ammonium nitrogen value in the control samples was significantly lower, at 4.8 mg N-NH_4_/L. When coffee extract was added at a concentration of 1 g/L, the concentration increased to 15.6 mg N-NH_4_/L. However, in samples with the addition of extract at a concentration of 10 g/L, a decrease to 2.3 mg N-NH_4_/L was observed, and for a concentration of 20 g/L, the value was 8.4 mg N-NH_4_/L.

The lowest ammonium nitrogen values were obtained with the phase II module at an extract concentration of 10 g/L (2.3 mg N-NH_4_/L). The use of the phase II module generally promoted higher ammonium nitrogen reduction efficiency compared to the phase I module, especially at medium coffee extract concentrations. The results confirm that the amount of coffee extract added and the configuration of the reactor’s operating phases significantly affect the efficiency of ammonium nitrogen reduction in treated wastewater.

**Figure 10:**
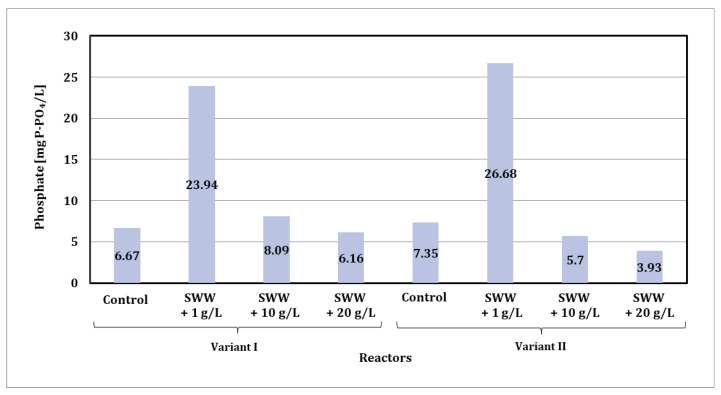
Phosphate values obtained after biological treatment of synthetic wastewater (SWW) and synthetic wastewater with coffee extract (own elaboration).

Based on the obtained test results, it was found that the phosphate content in wastewater after biological treatment in the SBR reactor varied depending on the composition of the wastewater fed into the reactors and the phase module used (Figure 10).

In the case of phase module I, the phosphate value after treatment was 6.67 mg P-PO_4_/L for synthetic wastewater. For wastewater with added coffee extract, these values were higher and amounted to 23.94 mg P-PO_4_/L at a concentration of 1 g/L, 8.09 mg P-PO_4_/L at a concentration of 10 g/L, and 6.16 mg P-PO_4_/L at a concentration of 20 g/L.

In the case of the phase II module, the phosphate content in synthetic wastewater was 7.35 mg P-PO_4_/L. For wastewater with added coffee extract, values of 26.68 mg P-PO_4_/L at a concentration of 1 g/L, 5.70 mg P-PO_4_/L at a concentration of 10 g/L, and 3.96 mg P-PO_4_/L at a concentration of 20 g/L were recorded.

The results indicate that the biological treatment process effectively reduced the phosphate content in all samples, although the degree of reduction depended on the composition of the wastewater and the phase module used. The highest phosphate content after treatment was observed in samples with the addition of coffee extract at a concentration of 1 g/L, which may be the result of a higher amount of easily biodegradable organic substances in this dose. Lower phosphate values in samples with higher extract concentrations (10 and 20 g/L) may suggest more effective phosphate removal, especially in the case of phase II. Additionally, it should be noted that the dark brown color of the wastewater with the addition of coffee extract, which was not completely removed during the biological treatment process, may have affected the accuracy of the measurements, making it difficult to precisely analyze the phosphate content in the samples.

### 3.2. Integration of Biological Wastewater Treatment with Activated Carbon Sorption Process

#### 3.2.1. Static Method

**Figure 11:**
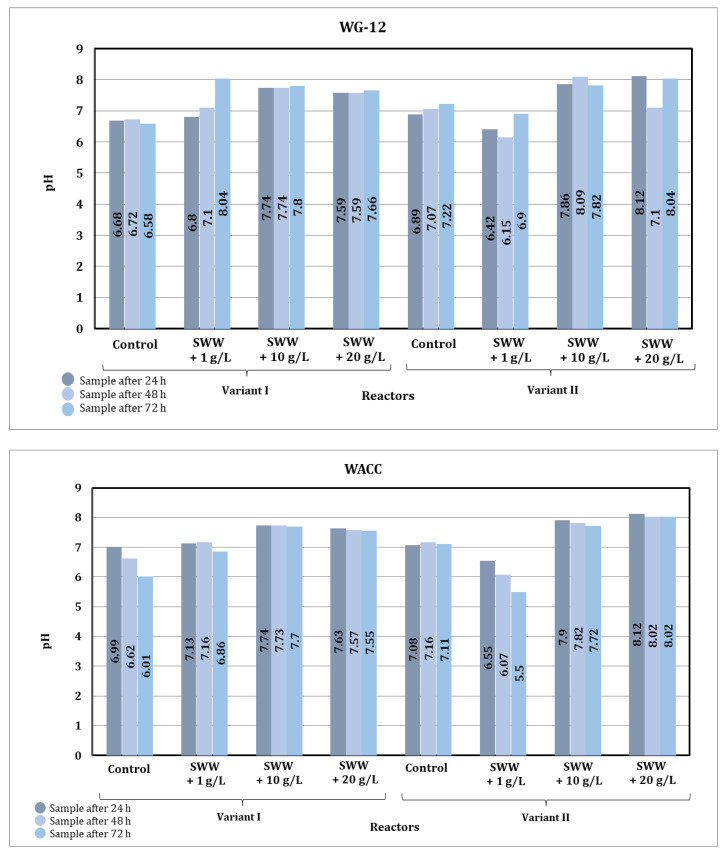
pH values obtained in the sorption process after 24, 48, and 72 h of contact with the selected sorption material (own elaboration).

Based on the results obtained, it was found that the pH value of biologically treated wastewater after the activated carbon sorption process underwent significant changes depending on the type of sorbent used, the contact time, the reactor operating phase module, and the composition of the wastewater introduced into the reactors (Figure 11).

The use of different types of activated carbon had an impact on the final pH value of the samples. Activated carbon known under the trade name WACC generally led to lower pH values compared to activated carbon known under the trade name WG-12, which may indicate its greater ability to adsorb ions, causing an increase in alkalinity. In particular, more stable pH values were observed in samples purified with WACC, especially in samples with the addition of coffee extract at concentrations of 10 g/L and 20 g/L.

The contact time between the sorbent and wastewater had a clear effect on the change in pH value. For both sorbents, a gradual decrease in pH value was observed in the control samples and in the samples with the addition of coffee extract at a concentration of 1 g/L as the contact time was extended to 72 h. In samples with higher extract concentrations (10 g/L and 20 g/L), the pH values were more stable, suggesting that the addition of organic substances limited further changes in pH during prolonged contact with activated carbon.

The differences in pH values were also related to the reactor operating phase module used. Phase II, characterized by alternating aeration and mixing, led to slightly higher pH values compared to phase I. This may be due to more effective mixing and better gas distribution, which favored the equilibration of the wastewater pH value.

The addition of coffee extract to synthetic wastewater had a significant effect on its pH value. In samples without the addition of extract, the initial pH value ranged from 4.91 to 5.85, indicating the acidic nature of synthetic wastewater. The addition of extract at a concentration of 1 g/L increased the pH value to between 5.09 and 5.44 before the sorption process, while at a concentration of 10 g/L the pH value ranged from 6.65 to 7.33, and at a concentration of 20 g/L it reached 7.28 to 7.45. After the sorption process on activated carbon, the pH values increased, which indicates the beneficial effect of the sorbent on the stabilization of the wastewater pH value.

The addition of higher concentrations of coffee extract (10 g/L and 20 g/L) limited pH value changes during the sorption process, which may be due to the buffering effect of the substances contained in the extract. In samples with lower extract concentrations (1 g/L) and in control samples, pH value changes were more pronounced, indicating lower resistance to the sorption process.

Analysis of the results indicates that the sorption process on activated carbon significantly stabilizes the pH value of the treated wastewater, especially when higher concentrations of coffee extract are added. The use of WG-12 and WACC activated carbon produced different effects depending on the type of wastewater being treated, the reactor phase module used, and the contact time of the sample with the sorption material. The Phase II module and longer contact time promoted higher pH value stabilization efficiency.

**Figure 12:**
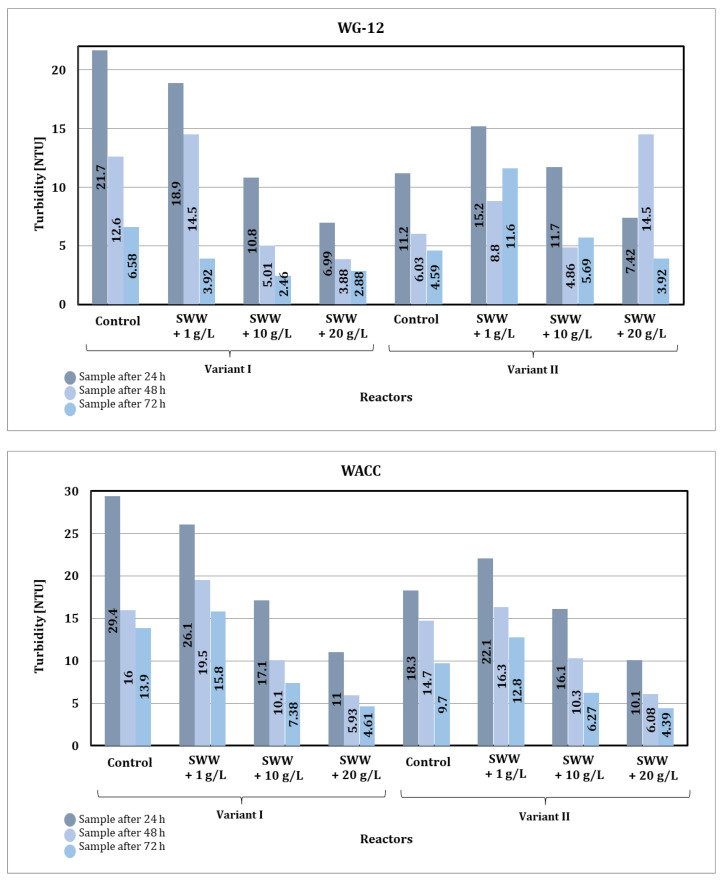
Turbidity values obtained in the sorption process after 24, 48, and 72 h of contact with the selected sorption material (own elaboration).

Based on the results obtained from measuring the turbidity of wastewater after the sorption process, significant changes were observed depending on the addition of coffee extract, the type of activated carbon used, and the contact time of the sample with the sorbent (Figure 12).

The addition of coffee extract to the treated synthetic wastewater had a clear effect on reducing wastewater turbidity during the sorption process. In the case of control samples that did not contain coffee extract, the initial turbidity values were the highest. After the sorption process, a significant reduction in turbidity was observed, but the final values were higher compared to samples with the addition of extract. In the case of samples with coffee extract at a concentration of 1 g/L, the initial turbidity was also high (32.2 and 32.5 NTU, respectively), but the sorption process resulted in a significant reduction in turbidity. Samples with extract concentrations of 10 g/L and 20 g/L had lower initial turbidity values (4.79–10.50 NTU), and the final values were the lowest among all samples, indicating the beneficial effect of higher extract concentrations on the efficiency of the sorption process.

The type of activated carbon used had a significant impact on the effectiveness of turbidity reduction. In the case of WG-12 activated carbon, better turbidity reduction results were observed compared to WACC, regardless of the reactor operating phase module and the concentration of the added coffee extract. For the control samples, the initial turbidity was reduced to 6.58 and 4.59 NTU, respectively, using WG-12 activated carbon after 72 h of contact, while for WACC, these values were 13.9 and 9.70 NTU, respectively. This difference indicates the higher effectiveness of WG-12 in removing suspended solids and fine particles responsible for turbidity.

The contact time of the sample with activated carbon was crucial for the effectiveness of the process. It was observed that the lowest turbidity values were obtained after 72 h of contact with the sorbent, regardless of the type of activated carbon. In the case of control samples, turbidity decreased systematically as the contact time increased. Similar trends were observed in samples with added coffee extract, although the differences between 48 and 72 h were less pronounced at higher extract concentrations, which may indicate faster saturation of the sorption surface of activated carbon.

The reactor phase module also had an impact on the turbidity measurement results. Samples treated in the phase II module (alternating aeration and mixing) generally showed lower turbidity values compared to the phase I module (intensive simultaneous aeration and mixing). For example, for a sample (1 g/L coffee extract, WACC), the turbidity after 72 h was 12.8 NTU in the phase II module, while in the phase I module it was higher at 15.8 NTU.

In summary, the use of WG-12 activated carbon resulted in lower turbidity values compared to WACC, and extending the contact time with the sorbent further increased the effectiveness of turbidity reduction. The phase II module, thanks to alternating aeration and mixing, proved to be more effective than the phase I module, especially in samples with high initial turbidity.

**Figure 13:**
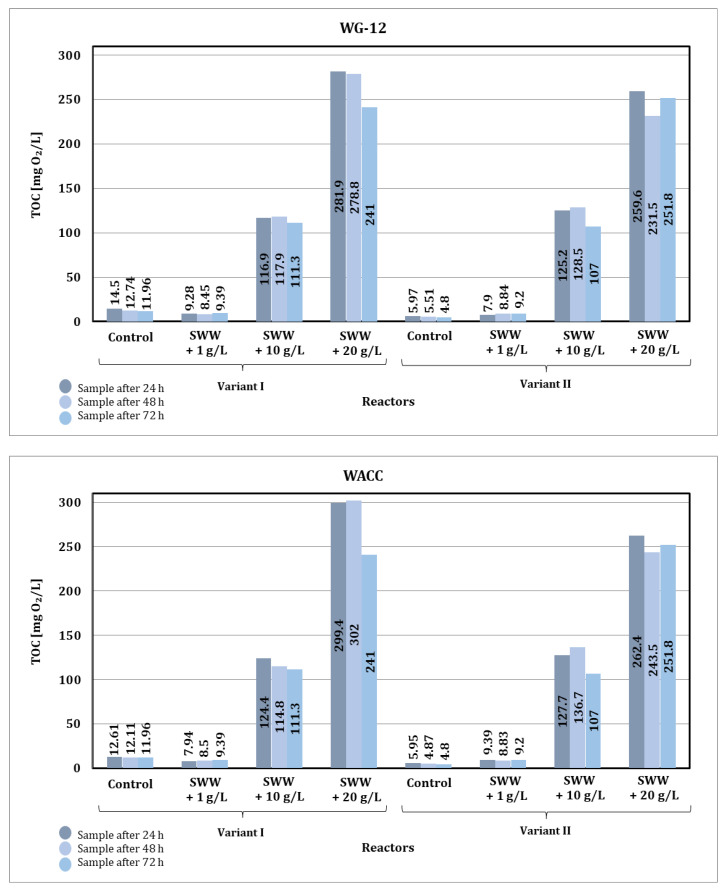
TOC values obtained in the sorption process after 24, 48, and 72 h of contact with the selected sorption material (own elaboration).

Analysis of the results (Figure 13) indicates that the addition of coffee extract to treated wastewater significantly affects the efficiency of TOC reduction in the sorption process. Synthetic wastewater without the addition of coffee extract had the lowest TOC values both before and after the sorption process, suggesting that it was easier to treat. The introduction of coffee extract at a concentration of 1 g/L resulted in an increase in TOC content compared to the control samples, but the sorption process was still relatively effective, especially when a longer contact time was used. At higher concentrations of coffee extract (10 and 20 g/L), the TOC values after biological treatment were significantly higher, indicating a higher organic load. This, in turn, reduced the effectiveness of the sorption process, especially in samples with the addition of coffee extract at a concentration of 20 g/L.

The type of activated carbon used also had a significant impact on the effectiveness of TOC reduction. WG-12 activated carbon showed better sorption properties than WACC, especially in control samples and those with lower coffee extract concentrations. Samples containing extract in higher concentrations (10 and 20 g/L) were more difficult to clean, and the effectiveness of WACC activated carbon was particularly limited in these cases.

The contact time between wastewater and sorbent significantly affected TOC reduction. Shorter contact times (24 h) yielded the least effective results in all analyzed samples. Extending the contact time to 48 h resulted in a significant improvement, especially in control samples and those with low coffee extract concentrations. The best results were obtained after 72 h of contact. However, even after this time, samples containing higher concentrations of coffee extract still showed high TOC content. This suggests that with a high organic load, even extending the contact time may not be sufficient for complete TOC reduction.

In summary, the addition of coffee extract significantly increases the organic load of wastewater, which makes it difficult to treat, especially at higher concentrations. WG-12 activated carbon is more effective in reducing TOC compared to WACC, especially in samples with a lower organic load. Extending the contact time is a key factor in improving the efficiency of the sorption process, although additional technological measures are necessary in the case of high extract concentrations.

**Figure 14:**
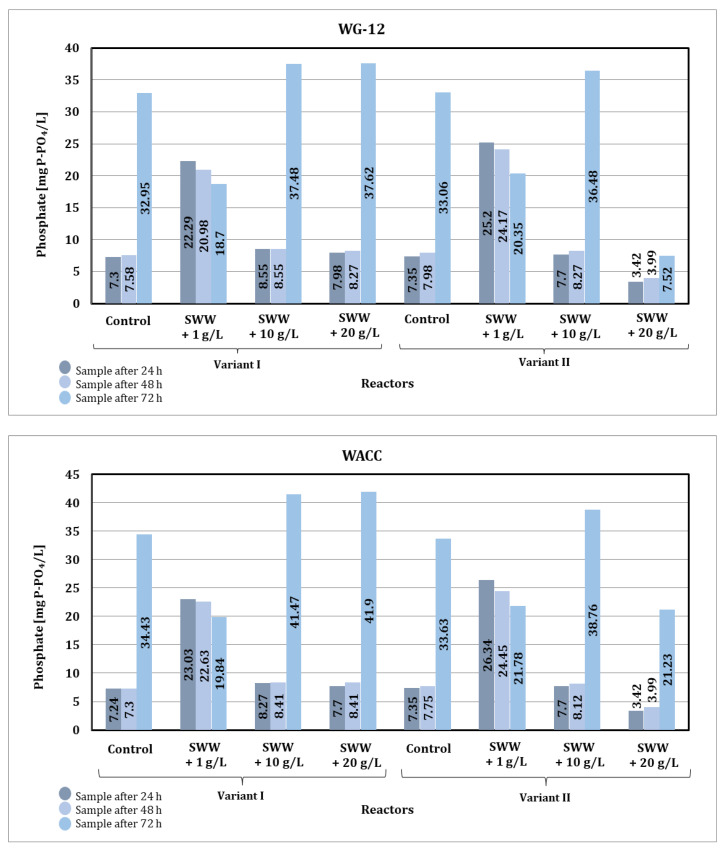
Phosphate values obtained in the sorption process after 24, 48, and 72 h of contact with the selected sorption material (own elaboration).

Analysis of the results (Figure 14) indicates that the addition of coffee extract to treated wastewater significantly affects the efficiency of phosphate reduction in the sorption process. Synthetic wastewater without the addition of coffee extract showed a stable level of phosphate reduction during the sorption process. In the case of samples containing coffee extract at a concentration of 1 g/L, a clear increase in the initial phosphate content was observed, which could have been due to the additional organic load introduced by the extract. The high phosphate content persisted throughout the treatment process, indicating difficulties in its removal in the presence of the extract.

Samples with higher concentrations of coffee extract (10 and 20 g/L) showed different trends. In some cases, the phosphate content after 24 h was lower than in the control samples, suggesting a possible interaction between the addition of coffee extract and the sorption mechanism. However, with longer contact time (72 h), the phosphate content in samples containing higher concentrations of extract increased significantly, which may have been due to the release of phosphorus from the decomposition of organic substances contained in the coffee extract.

The type of activated carbon used also affected the efficiency of the process. WG-12 activated carbon showed better sorption properties in samples with lower initial phosphate loads (e.g., control samples and those with lower coffee extract concentrations). WACC activated carbon was less effective, especially in samples with high phosphate content, which may indicate its lower ability to bind phosphates in the presence of organic compounds contained in coffee extract.

The contact time between the sample and activated carbon also affected changes in phosphate content. Shorter times (24 and 48 h) allowed for a certain reduction in phosphate levels in most samples, but after 72 h, a significant increase in phosphate content was observed, especially in control samples and those with higher concentrations of coffee extract. A possible explanation for this phenomenon is the desorption of previously bound phosphates or their release as a result of the decomposition of organic matter. This process may have been further enhanced by changes in the pH of the system.

In summary, the addition of coffee extract to synthetic wastewater significantly increases the initial phosphate load, which limits the effectiveness of sorption treatment, especially at higher extract concentrations. WG-12 activated carbon was more effective in binding phosphates in samples with lower loads, while WACC activated carbon showed limited sorption capacity. Extending the contact time to 72 h in many cases led to an increase in phosphate content, which may be related to the release of phosphorus from the decomposition of organic compounds or the desorption of previously bound phosphates. To increase the efficiency of the process, it is necessary to integrate the sorption process with other technologies.

#### 3.2.2. Dynamic Method

**Figure 15:**
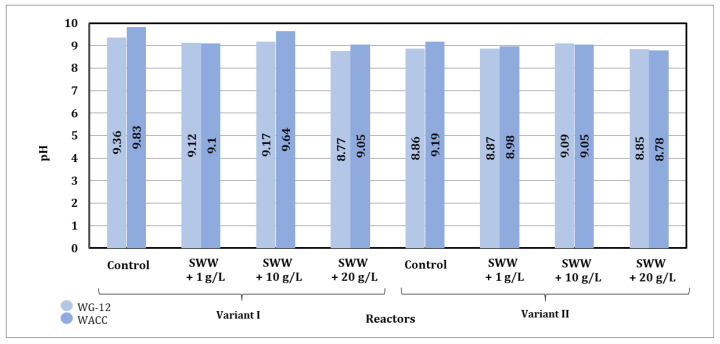
pH values obtained in the sorption process for two sorption materials (own elaboration).

Based on the results obtained, it was found that the pH value obtained after the sorption process on activated carbon underwent significant changes depending on the type of sorbent used and the chemical composition of the wastewater subjected to the process (Figure 15).

The final pH values after the sorption process under dynamic conditions were comparable for both activated carbons used, which may indicate a similar sorption capacity of these materials under the analyzed process conditions.

After the sorption process under dynamic conditions, the pH values increased significantly more than in the case of sorption under static conditions. In samples without the addition of extract, pH values ranging from 8.86 to 9.83 were obtained after the sorption process. In contrast, in samples to which coffee extract at a concentration of 1 g/L had been previously added, pH values ranging from 8.87 to 9.12 were obtained after the sorption process, at a concentration of 10 g/L, pH values ranging from 9.05 to 9.64 were obtained, and at a concentration of 20 g/L, pH values ranging from 8.77 to 9.05 were obtained.

**Figure 16:**
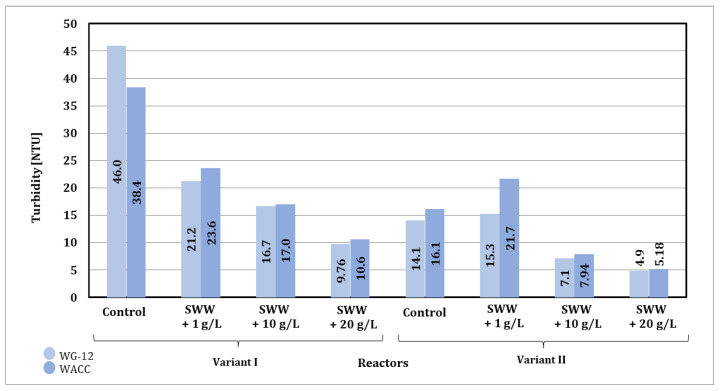
Turbidity values obtained in the sorption process for two sorption materials (own elaboration).

Based on the results obtained from measuring the turbidity of wastewater after the sorption process, significant changes were observed depending on the concentration of the previously applied coffee extract additive and the type of activated carbon used (Figure 16).

The reactor phase module also had an impact on the turbidity measurement results. For samples treated in phase II (alternating aeration and mixing), lower turbidity values were obtained compared to phase I (intensive simultaneous aeration and mixing).

The type of activated carbon used also had a significant impact on the effectiveness of turbidity removal. In the case of WG-12 activated carbon, better turbidity removal results were observed compared to WACC, regardless of the reactor phase module and the concentration of the added coffee extract. When WG-12 activated carbon was used for samples without the addition of extract, turbidity levels of 46.0 NTU (phase I module) and 14.1 NTU (phase II module) were obtained after the sorption process. However, for samples to which coffee extract had previously been added at a concentration of 1 g/L, turbidity levels of 21.2 NTU (phase I module) and 15.3 NTU (phase II module) were obtained, while at a concentration of 10 g/L turbidity levels of 16.7 NTU (phase I module) and 7.1 NTU (phase II module), and at a concentration of 20 g/L, turbidity levels of 9.76 NTU (phase I module) and 4.9 NTU (phase II module) were obtained.

In summary, as in the case of sorption under static conditions, the use of WG-12 activated carbon resulted in lower turbidity values compared to WACC. The phase II module, thanks to alternating aeration and mixing, proved to be more effective than the phase I module, especially in samples with high initial turbidity. The turbidity values obtained after sorption under static (72 h of contact) and dynamic conditions were largely comparable in all cases analyzed. Considering the results obtained and the economic aspect, it is more advantageous to use dynamic sorption in an integrated technological process.

**Figure 17:**
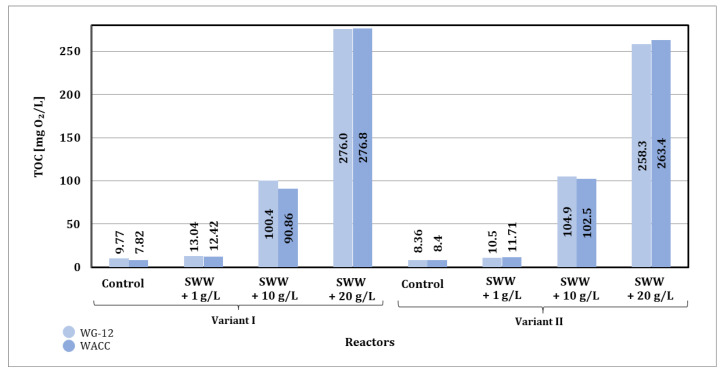
TOC values obtained in the sorption process for two sorption materials (own elaboration).

Based on the obtained results of TOC values of wastewater after the sorption process, significant changes were also observed depending on the concentration of the previously used coffee extract additive and the type of activated carbon used (Figure 17).

The final TOC values after the sorption process under dynamic conditions were comparable for both activated carbons used, which may indicate their comparable sorption capacity under the analyzed process conditions.

In summary, the TOC values obtained after sorption under static (72 h of contact) and dynamic conditions were largely comparable in all cases analyzed. Considering the TOC results obtained and the economic aspect of both analyzed conditions of the sorption process, it is more advantageous to use dynamic conditions in the selected integrated sorption process, mainly due to the significantly shorter process time. The optimization of process parameters, such as the height and width of the sorption bed filling the filter column, is a key factor that can improve the efficiency of the sorption process.

**Figure 18:**
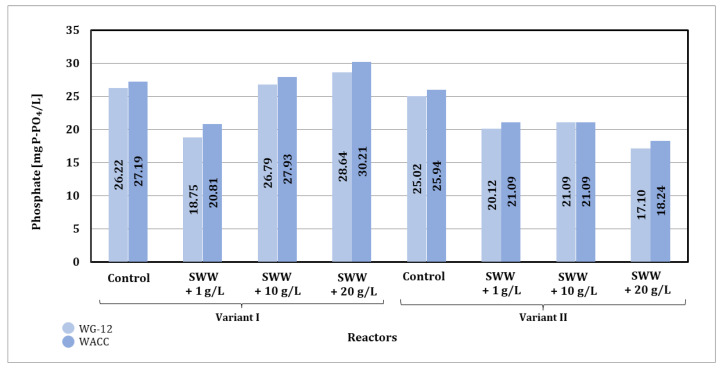
Phosphate values obtained in the sorption process for two sorption materials (own elaboration).

Based on the results obtained for phosphate values in samples after the sorption process, significant changes were observed depending on the concentration of the previously applied coffee extract additive and the type of activated carbon used (Figure 18).

The type of activated carbon used also had a significant impact on the effectiveness of phosphate reduction. In the case of WG-12 activated carbon, better phosphate reduction results were observed compared to WACC, regardless of the reactor operating phase module and the concentration of the added coffee extract. When WG-12 activated carbon was used for samples without the addition of extract, the phosphate value after the sorption process was 26.22 mg P-PO_4_/L (phase I module) and 25.02 mg P-PO_4_/L (phase II module). However, for samples to which coffee extract had previously been added at a concentration of 1 g/L, the phosphate value obtained was 18.75 mg P-PO_4_/L (phase I module) and 20.12 mg P-PO_4_/L (phase II module), while at a concentration of 10 g/L the phosphate value was 26.79 mg P-PO_4_/L (phase I module) and 21.09 mg P-PO_4_/L (phase II module), and at a concentration of 20 g/L, the phosphate value obtained was 28.64 mg P-PO_4_/L (phase I module) and 17.19 mg P-PO_4_/L (phase II module).

In summary, the phosphate values obtained after sorption under static conditions (72 h of contact) were higher than those obtained under dynamic conditions. Considering the results obtained and the economic aspect of both analyzed conditions of the sorption process, it is more advantageous to use dynamic sorption in the selected integrated technological line.

Analysis of sorption results under static and dynamic conditions showed higher efficiency and shorter contact time in the dynamic process. The dynamic method, involving the flow of the sample through the activated carbon bed, provided better reduction in turbidity, TOC, and phosphates, while demonstrating greater operational and economic efficiency. Due to these advantages, sorption under dynamic conditions was selected for further research.

### 3.3. Integration of Biological Wastewater Treatment with Sorption and Membrane Filtration Processes

**Figure 19:**
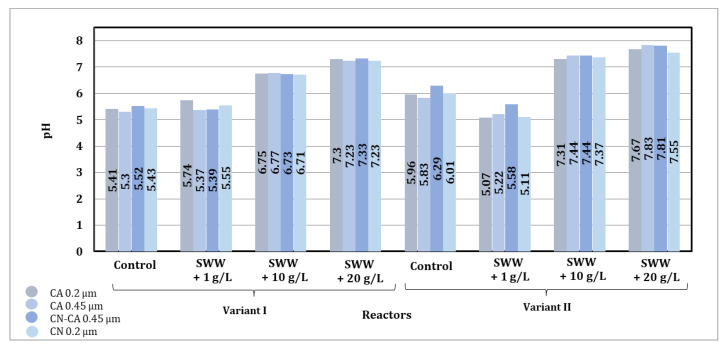
pH values obtained in the membrane filtration process for four different membranes (own elaboration).

Based on the test results, it was found that the pH values obtained after the filtration process were comparable for all membranes used, which may indicate their comparable filtration capacity (Figure 19).

**Figure 20:**
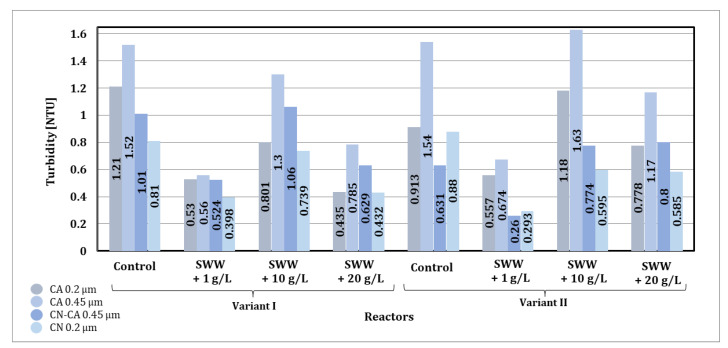
Turbidity values obtained in the membrane filtration process for four different membranes (own elaboration).

Based on the test results, it was found that the highest reduction in turbidity after filtration was achieved when using a CN membrane with a pore diameter of 0.2 µm (Figure 20). The CN 0.2 µm membrane showed the best turbidity reduction results compared to the other membranes analyzed, regardless of the concentration of the previously added coffee extract. When using the CN 0.2 µm membrane, turbidity below 0.8 NTU was recorded in all analyzed samples.

In summary, cellulose nitrate membranes with a pore diameter of 0.2 µm proved to be effective in membrane filtration for wastewater treatment due to their ability to retain contaminants while maintaining high filtrate quality. In addition, these membranes are relatively inexpensive and easy to manufacture, making them an attractive solution for wastewater treatment plants. Therefore, it was decided that CN 0.2 µm membranes would be the most advantageous for further research.

### 3.4. Multi-Stage Wastewater Treatment with Disinfection

Biological wastewater treatment effectively reduces key pollutants, but its effects often fail to meet applicable environmental standards. Therefore, based on the above-mentioned studies, the most favorable process conditions were selected from among the analyzed variants, and the integrated multi-stage technological sequence was repeated, including sorption under dynamic conditions on WG-12 activated carbon, membrane filtration with cellulose nitrate membranes with a pore diameter of 0.2 µm, and disinfection by ozonation (20 min) and UV radiation (20 min).

**Figure 21:**
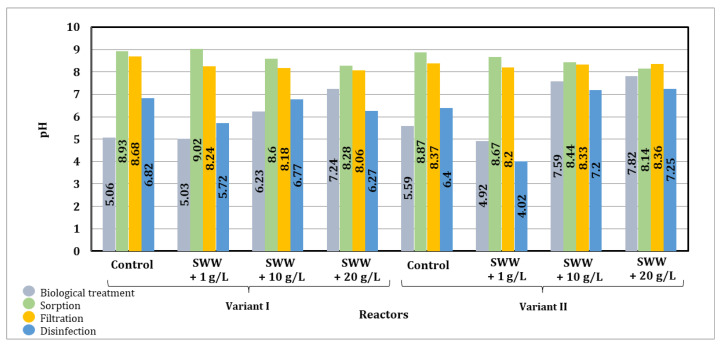
pH value obtained after each stage of wastewater treatment and disinfection (own elaboration).

Based on the results presented in Figure 21, it was noted that after biological treatment, the pH value of samples without the addition of coffee extract decreased from 6.43 to 5.06–5.59. Similarly, a decrease in pH was observed in samples to which coffee extract had previously been added at a concentration of 1 g/L from 6.77 to 4.92–5.03 after the process. On the other hand, an increase in pH was observed in samples to which extract had previously been added at a concentration of 10 g/L, from 5.76 to 6.23–7.59. Similarly, in samples to which an extract at a concentration of 20 g/L had been previously added, the pH value increased from 5.64 to 7.24–7.82. After the sorption process, the pH value increased in all samples, which could have been the result of the alkalizing effect of the activated carbon used. Membrane filtration caused only a slight decrease in pH, as this process is limited to the physical separation of contaminants without significant chemical changes. After disinfection of the samples, a stronger decrease in pH value was observed. In samples without the addition of extract, the pH value was similar to that before treatment, while in samples with an extract concentration of 1 g/L, it was lower. For concentrations of 10 and 20 g/L, the pH value after disinfection was higher than before the treatment process, which may have been due to the presence of organic compounds contained in the coffee extract, affecting the acid–base balance.

The results of the study indicate that integrated, multi-stage wastewater treatment allowed for controlled pH adjustment, enabling the production of treated wastewater with stable and desirable physicochemical properties, which is crucial for its further use.

**Figure 22:**
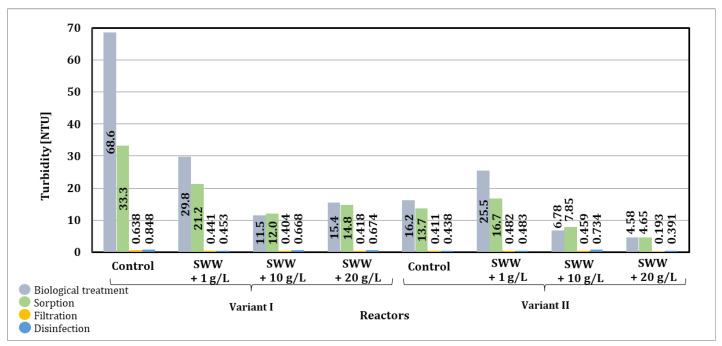
Turbidity value obtained after each stage of wastewater treatment and disinfection (own elaboration).

Based on the results presented in Figure 22, it can be seen that membrane filtration is a key stage in reducing turbidity. This stage allowed for a final reduction in turbidity in all analyzed samples in the range from 90 to even 99%. The test results confirmed that membrane filtration proved to be justified in the integrated technological line used, as it is limited to the physical separation of contaminants, thus minimizing chemical changes. This makes the process more environmentally friendly and allows it to be used in various stages of treatment, including advanced processes where high-quality recovered water is required.

**Figure 23:**
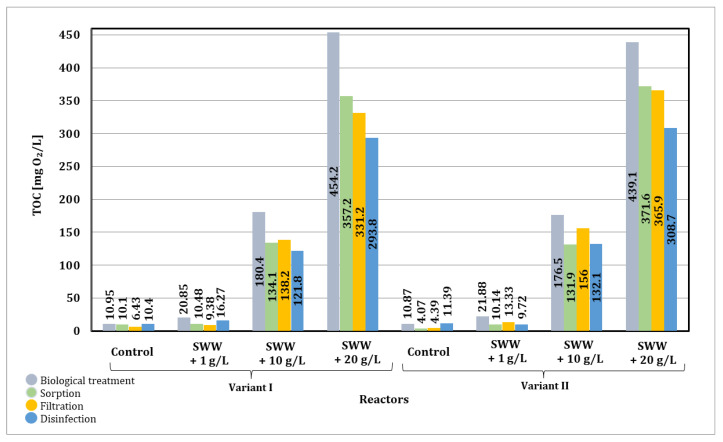
TOC value obtained after each stage of wastewater treatment and disinfection (own elaboration).

Based on the results presented in Figure 23, it was noted that after biological treatment, the greatest reduction in TOC was observed in all samples. This indicates the important role of biological treatment in reducing key pollutants. The TOC value in samples without the addition of coffee extract decreased from 82.64 mg O_2_/L to 10.87–10.95 mg O_2_/L. Similarly, a decrease in TOC values was observed in samples to which extract had previously been added at a concentration of 1 g/L from 212.1 mg O_2_/L to 20.85–21.88 mg O_2_/L. In samples to which an extract at a concentration of 10 g/L had previously been added, there was a decrease in TOC values from 922.2 mg O_2_/L to 176.5–180.4 mg O_2_/L. Similarly, in samples to which an extract at a concentration of 20 g/L had been previously added, there was a decrease in TOC values from 1750.0 mg O_2_/L to 439.1–454.2 mg O_2_/L. After the treatment and disinfection processes, TOC values decreased in all samples.

The integrated multi-stage technological process enabled a final reduction in TOC values in samples without the addition of coffee extract of 86–87%. A higher final reduction in TOC values was observed in samples to which coffee extract had previously been added at a concentration of 1 g/L, reaching 92–95%. A slightly lower final reduction was observed in samples to which extract had previously been added at a concentration of 10 and 20 g/L, ranging from 82–87%. The multi-stage process used allowed for the effective removal of TOC as well as other key contaminants. The stages selected for the process worked synergistically, allowing for the removal of various types of contaminants.

**Figure 24:**
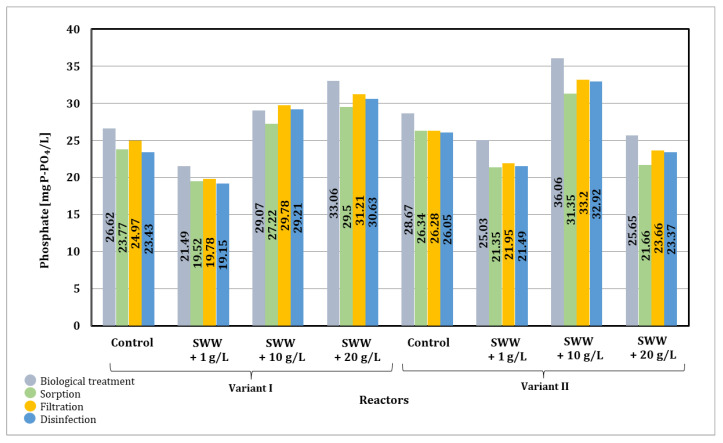
Phosphate value obtained after each stage of wastewater treatment and disinfection (own elaboration).

Based on the results presented in Figure 24, it was noted that the process parameters of the integrated multi-stage technological line require further optimization to effectively reduce phosphates in the samples. The key stage requiring optimization is the biological wastewater treatment stage. The test results clearly indicate that modifying the duration of individual SBR phases may enable effective reduction of biogenic compounds, although this issue requires further in-depth research.

A decrease in final phosphate values of 27–47% was observed in samples to which coffee extract at a concentration of 1 g/L had been previously added. In samples to which extract at a concentration of 20 g/L had been previously added, only in phase II (alternating aeration and mixing) was a final reduction in phosphate levels of 16% observed.

It should be emphasized that, in the case of control wastewater (without coffee extract), the concentration of phosphates after biological treatment was noticeably higher than before the process. This phenomenon can be explained by the release of phosphorus from activated sludge biomass (so-called phosphorus release), which may occur under non-optimal operating conditions, particularly in the absence of clearly separated anaerobic and aerobic phases required for effective enhanced biological phosphorus removal. Additionally, the mineralization of organic phosphorus compounds present in the wastewater may have contributed to an increase in orthophosphate concentrations.

In contrast, in wastewater containing coffee extract, a significantly smaller difference was observed between phosphate concentrations before and after biological treatment. This is because the initial phosphate levels in these samples were already higher, likely due to the presence of phosphorus compounds in the coffee extract. After biological treatment, only a slight increase in phosphate concentration was observed, which may indicate partial stabilization of phosphorus transformations in the system and a different interaction between biomass and organic substrates.

Furthermore, the subsequent treatment stages (sorption, membrane filtration, and disinfection) were not highly effective in phosphate removal. This is mainly because phosphates are present in dissolved form (orthophosphates), which are not easily removed by physical separation processes such as membrane filtration, nor are they effectively adsorbed under the applied conditions without prior chemical precipitation. Similarly, ozonation and UV radiation are primarily aimed at disinfection and oxidation of organic compounds and do not significantly contribute to phosphorus removal.

These results indicate that the observed variability and relatively low efficiency of phosphate removal are primarily related to the limitations of the biological stage and the lack of dedicated phosphorus removal mechanisms (e.g., chemical precipitation), highlighting the need for further process optimization.

**Figure 25:**
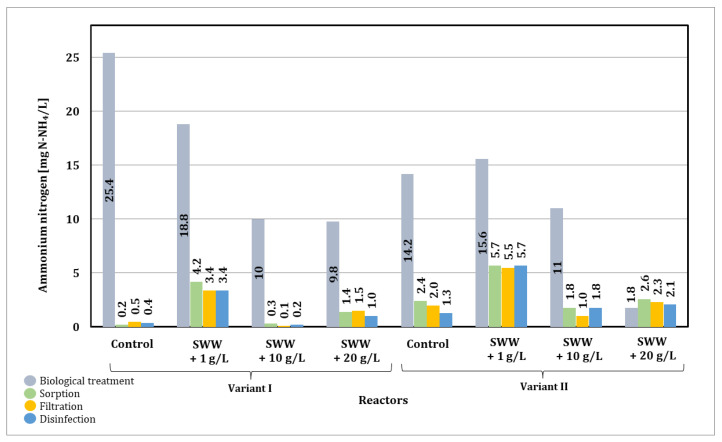
Ammonium nitrogen value obtained after each stage of wastewater treatment and disinfection (own elaboration).

The results presented in Figure 25 indicate that in order to effectively reduce biogenic compounds such as phosphorus and nitrogen, the key stage requiring optimization is the biological wastewater treatment stage. The results of the study clearly indicate that changing the duration of individual SBR phases can enable effective reduction of biogenic compounds.

A greater reduction in ammonium nitrogen was achieved with the phase I module (continuous mixing and aeration). The integrated multi-stage process enabled a final reduction in ammonium nitrogen in samples without the addition of coffee extract of 86–96%. The lowest final reduction in ammonium nitrogen was in samples to which coffee extract had previously been added at a concentration of 1 g/L at 38%. A significantly higher final reduction was observed in samples to which extract had been previously added at concentrations of 10 and 20 g/L, at 73–97% and 77–89%, respectively.

It should be emphasized, however, that after the biological treatment stage, in most of the analyzed samples, the concentration of ammonium nitrogen was higher than in the raw wastewater. This phenomenon may be explained by the mineralization (ammonification) of organic nitrogen compounds present in the wastewater, leading to the release of ammonium ions. At the same time, incomplete nitrification—resulting from suboptimal oxygen conditions and/or insufficient adaptation of activated sludge microorganisms to the presence of coffee-derived compounds—limited the further oxidation of ammonium nitrogen to nitrates.

A noticeable reduction in ammonium nitrogen was observed only after the sorption stage, which indicates that activated carbon played a significant role in removing nitrogen compounds, most likely through adsorption of ammonium ions and organic nitrogen fractions. This confirms that the biological stage alone was insufficient and that the subsequent processes in the integrated system were necessary to achieve satisfactory removal efficiency.

The observed variability in ammonium nitrogen removal efficiency can therefore be attributed to the combined effects of: transformation of organic nitrogen into ammonium during biological treatment, differences in reactor operating modes (phase configuration), and the influence of wastewater composition, particularly the presence of organic compounds in coffee extract that may inhibit nitrification processes.

The use of biological wastewater treatment alone did not allow for the effective removal of ammonium nitrogen. It was necessary to use a multi-stage technological process, where selected stages worked synergistically, allowing for the removal of various types of pollutants.

In light of the research conducted, it was found that despite the use of an integrated technological line, not all of the analyzed physicochemical parameters (pH, turbidity, total organic carbon, ammonium nitrogen, phosphates) met the requirements of applicable environmental standards for the discharge of wastewater into the environment and its reuse. Although the individual stages of the process significantly reduced pollutant concentrations, further adjustment of the technological parameters is necessary to achieve full compliance with the requirements. In particular, the results indicate the need for optimization of the biological treatment stage, which plays a decisive role in the removal of biogenic compounds, as well as the potential incorporation of additional dedicated processes (e.g., enhanced nutrient removal or chemical precipitation) to improve overall system performance.

However, the technological line used shows high potential for achieving the set goals. The obtained results confirm the synergistic effect of combining biological treatment, sorption, membrane filtration, and advanced oxidation/disinfection processes, especially in terms of organic matter and turbidity removal. Further research is needed to optimize individual processes, including the configuration and duration of SBR phases, operating conditions of sorption and membrane filtration, and parameters of ozonation and UV radiation, in order to improve the efficiency of removing more recalcitrant pollutants characteristic of wastewater from coffee processing.

In addition, coffee extract, which served as a model pollutant in the studies, highlights the unique challenges associated with treating wastewater from the coffee industry. Its specific chemical composition, including high concentrations of organic substances, the presence of potentially inhibitory compounds (e.g., phenolics, caffeine), and relatively low pH, hinders effective treatment using standard methods. At the same time, the variability in pollutant load further complicates process stability and efficiency. These findings confirm the need for integrated and carefully optimized treatment technologies capable of addressing the complex and variable nature of this type of wastewater.

## 4. Discussion

Studies conducted on model wastewater containing coffee bean extract have shown that the use of integrated wastewater treatment processes, including biological treatment, activated carbon sorption, membrane filtration, and two-stage disinfection using ozone and UV radiation, has significant potential to achieve high efficiency in removing organic, biogenic, and microbiological pollutants that are critical to safety. The individual stages of the treatment process are discussed in detail below, comparing them with the literature and indicating potential limitations and opportunities for optimization.

### 4.1. Biological Treatment

The studies conducted showed that the addition of a model pollutant, namely coffee bean extract, had a significant impact on the biological wastewater treatment process. Among other things, an increase in pH value and a marked reduction in turbidity were observed in samples with higher doses of the extract, indicating the effective adaptation of activated sludge microorganisms to the presence of compounds derived from coffee bean extract. In turn, the total organic carbon content in samples with the addition of extract increased significantly with an increase in the concentration of the model pollutant, suggesting that the substances present in the extract were an additional, difficult-to-biodegrade source of carbon. Previous studies conducted under conditions very similar to those presented in this paper [23], where caffeine was used as a model pollutant, confirm that the treatment of wastewater from coffee processing is a significant problem. Caffeine itself, present in coffee processing wastewater, is not a significant problem in the proposed integrated technological process, but other numerous substances present in coffee bean extract, which act synergistically, pose a challenge. This is confirmed by a comparison of the results presented in the study with the results obtained from previous research, in which synthetic wastewater with the addition of pure caffeine was used during the biological treatment process in SBRs, revealing both similarities and differences. Previous studies have shown that the presence of caffeine promoted increased microbial activity, which resulted in a high degree of TOC removal (86–91%) and ammonium nitrogen removal (41–63%). In our own studies using coffee bean extract, the degree of TOC removal under the most favorable process conditions was significantly lower (69.3–88.5%), especially at higher concentrations of the model pollutant additive, while the reduction in ammonium nitrogen concentration in many tests was more varied (0–65.2%). It can be assumed that caffeine is a more easily assimilable source of carbon for microorganisms than the complex mixture of compounds present in coffee bean extract. Similarly, concerning phosphates, both studies achieved an effective removal rate, although in the case of wastewater with extract, the differences were strongly dependent on the concentration of the additive and the reactor operating variant—under the most favorable process conditions, a removal rate of 25.4–85.9% was achieved. In contrast, in tests with caffeine, the efficiency of phosphate removal was more stable, reaching 64–65% [23]. In summary, the results obtained indicate that both pure caffeine and coffee bean extract affect the activity of activated sludge, but the mechanisms of this effect are different. Caffeine can act as an additional substrate, increasing the metabolism of microorganisms, while coffee bean extract also introduces other bioactive substances into the environment, which in some cases can inhibit the biodegradation process.

Numerous data in the literature confirm the above assumption that the complex composition of wastewater from coffee processing poses a significant challenge. A review of available wastewater treatment techniques for the coffee industry shows that, due to the presence of phenolic compounds, alkaloids, and dyes, standard biological processes often have limited effectiveness. An analysis conducted by Ijanu et al. (2020) [30] confirmed the limited removal of key pollutants in coffee processing wastewater using a standard biological treatment process. Biological treatment is commonly used to remove BOD/COD, but it proves to be ineffective in eliminating color and acidic components of coffee wastewater, and these processes require a long time. Various methods are used, including aerobic and anaerobic treatment, activated sludge, enzymatic processes, and bioreactors, which allow for the removal of 50–80% of COD, depending on operating conditions. One of the key factors affecting the efficiency of biodegradation is the proper selection of the hydraulic retention time, the inaccurate estimation of which can lead to a reduction in treatment efficiency [31]. Another key factor affecting the efficiency of the process is the selection of appropriate reactor operating conditions. In their research, Chowaniec and Fryźlewicz-Kozak (2014) [32] showed that the intensity and duration of mixing in an SBR reactor affect the morphology of activated sludge and the efficiency of the process—longer aeration promotes the formation of more compact structures and a reduction in COD values, but if it is too long, it can cause floc fragmentation and impaired sedimentation. A similar relationship can be observed in the case of wastewater containing coffee bean extract, where the appropriate mixing time promotes the effective biodegradation of organic compounds.

The selection of appropriate operating conditions in the biological wastewater treatment process is crucial for treatment efficiency, but it does not always enable the removal of color from wastewater from coffee processing. An interesting alternative solution to this problem was proposed by Mahesh et al. (2014) [33] in their research, where they used preliminary treatment by electrocoagulation at a voltage of 35 V and a time of 45 min. This process enabled the removal of approximately 93% of the color and a significant reduction in COD and BOD. Although the total reduction in organic pollutants exceeded 85%, compounds affecting the color and conductivity of the wastewater remained. The results indicate that biological treatment combined with electrocoagulation is effective but insufficient for the complete treatment of wastewater from coffee processing. Therefore, additional processes are necessary.

Despite certain limitations, biological wastewater treatment remains a widely used and environmentally friendly alternative to chemical processes. Although chemical methods enable rapid decomposition of most organic pollutants, their use results in the formation of sludge containing reagent residues, which can pose an environmental threat. In biological processes, microorganisms break down organic compounds into simple mineral components without the need for chemicals, and in the case of wastewater containing coffee bean extract, this method has proven particularly effective in reducing organic carbon load and producing sludge with potential fertilizing properties [1]. However, it should be emphasized that this process is time-consuming, as microorganisms need time to adapt to the new environment before they can begin to effectively degrade organic compounds. An additional difficulty is the variability in the volume and concentration of wastewater, which can slow down the process and reduce its efficiency. To meet applicable environmental standards, the resulting wastewater requires additional final treatment stages [30].

### 4.2. Integration of Biological Wastewater Treatment with Activated Carbon Sorption Process

Sorption on activated carbon is one of the most commonly used methods of treating wastewater from the food industry, including dairy, confectionery, and oil production, due to its high efficiency in removing organic compounds and heavy metal ions. Literature data indicate that activated carbon, thanks to its large specific surface area, effectively adsorbs organic substances, phenols, and compounds that affect the turbidity and conductivity of wastewater. However, the effectiveness of sorption depends on the dose of sorbent, the type of material used, and the nature of the wastewater [34,35].

Although adsorption is considered a highly effective method for the removal of various contaminants, including organic compounds and micropollutants, its performance as a stand-alone process is limited. As reported by Rashid et al. [21], adsorption efficiency depends strongly on operational parameters and adsorbent characteristics, and the process may become economically unfavorable due to the need for adsorbent regeneration or replacement. Moreover, complete removal of pollutants is not always achieved, particularly in complex wastewaters with high organic loads, such as those generated by the coffee processing industry [30,34].

In contrast, biological treatment methods are widely recognized as cost-effective and efficient for the removal of biodegradable organic matter. For example, Shanmugam and Gummadi [25] demonstrated effective degradation of synthetic coffee wastewater using microbial cultures, confirming the high potential of biological processes in reducing organic load. However, biological treatment alone may be insufficient for the removal of more recalcitrant compounds or specific contaminants such as caffeine and other emerging pollutants, which can persist in treated effluents [23,31].

For this reason, recent studies increasingly emphasize the advantages of integrated and dynamic treatment systems. Ijanu et al. [30] and Aderibigbe et al. [34] highlight that combining different treatment methods allows for the complementary removal of pollutants with varying physicochemical properties. In particular, coupling biological processes with adsorption enables initial biodegradation of easily degradable compounds, followed by polishing of the effluent through adsorption, which improves overall treatment efficiency.

This approach is consistent with the findings reported by Skorupa et al. [23], where integrated systems combining biological treatment with additional processes such as adsorption and membrane filtration resulted in enhanced removal of contaminants, including caffeine. Similarly, studies on combined systems involving sequential operations (e.g., electrochemical treatment followed by biological reactors) indicate that process integration improves treatment performance compared to single-stage systems [33]. Additionally, operational factors such as mixing conditions can further influence the efficiency of treatment processes, particularly in dynamic systems, as noted by Chowaniec and Fryźlewicz-Kozak [32].

In the tests conducted, after the biological treatment stage, further wastewater treatment was necessary. The use of activated carbon sorption allowed for additional removal of TOC and biogenic substances, reduced turbidity, and contributed to pH stabilization. Analyses showed that the effectiveness of removing key pollutants depended on the type of sorbent used, the process method, and the model pollutant concentration. For both types of activated carbon (WG-12 and WACC), a further reduction in TOC, phosphates, and turbidity was observed, with better results obtained for WG-12 carbon. More favorable and repeatable results were also obtained in the dynamic method variant. Nevertheless, in tests with higher concentrations of coffee bean extract, the effectiveness of contaminant removal was limited, indicating that the compounds present in the extract may have reduced the sorption capacity of the sorbent.

The results obtained differed from those of earlier studies [23], in which pure caffeine was used as the model contaminant. In those studies, where identical sorption materials were used, WACC activated carbon achieved very high efficiency—TOC removal was 67–89%, ammonium nitrogen 75–81%, and turbidity 75–77%. In contrast, WG-12 activated carbon showed lower efficiency, achieving TOC removal of 34–60% and ammonium nitrogen removal of 7–14%. Interestingly, in tests with pure caffeine, the sorption efficiency was higher than in the control tests, suggesting that caffeine may promote the sorption process by increasing the ability of activated carbon to bind contaminants.

Although activated carbons are highly effective in removing organic and inorganic pollutants, their widespread use in industrial practice is often limited by high production and regeneration costs. The price of commercial activated carbons is mainly due to the cost of raw materials and energy-intensive physical or chemical activation processes. For this reason, there is growing interest in finding cheaper, more readily available, and more sustainable alternatives that could replace traditional sorbents without losing their effectiveness. The use of alternative activated carbons obtained from waste materials is a promising prospect for further research. The inclusion of such a sorbent in the developed technological process may not only reduce operating costs, but also increase the efficiency of the process [4]. The use of waste materials as a source of activated carbon is also in line with the principles of the circular economy, supporting sustainable development and reducing environmental impact [1]. There are numerous reports in the literature on activated carbons obtained from coffee waste or other agricultural waste [4]. In their study, Aouay et al. (2024) [36] showed that carbon from used coffee grounds, after phosphate activation, removed up to 98% of dyes under optimal conditions. Similar studies were conducted by Mayasopa et al. (2024) [37], who studied activated carbon from coffee husks in the treatment of food wastewater, achieving 88% BOD removal and 25% COD removal, which indicates that the effectiveness of the process depends on the type of pollutants and their concentration. In their research, Blinová and Sirotiak (2021) [38] also confirmed the effectiveness of adsorption in removing pollutants from wastewater, highlighting the potential of inexpensive adsorbents from industrial waste, such as burnt coffee grounds, which are characterized by high carbon content and numerous functional groups that bind organic compounds. An interesting solution was the use of avocado peel to produce activated carbon, which was then used to treat wastewater from a coffee processing plant. The resulting sorbent showed very high efficiency—the maximum COD reduction was 98.20% and that for BOD 99.18%; these results were comparable to the results for commercial carbon (99.02% and 99.35%) [39]. Equally interesting results were presented by Phan et al. (2019) [40], who described a new TRI-ARHA material obtained from rice husk ash. Its maximum adsorption capacity for nitrates was 163.4 mg/g. In addition, this material retained high stability and efficiency even after ten regeneration cycles, making it a promising solution for removing anions from wastewater [21].

### 4.3. Integration of Biological Wastewater Treatment with Sorption and Membrane Filtration Processes

In our own research, the use of membrane filtration after the activated carbon sorption stage significantly improved the quality of treated wastewater, constituting a key element in the integrated technological system. Cellulose nitrate (CN) membranes with a pore diameter of 0.2 µm proved to be the most effective, characterized by high resistance to fouling and stability over time. The use of these membranes made it possible to significantly reduce turbidity, confirming their effectiveness in removing both organic compounds and colloidal substances. The filtrate obtained was characterized by high transparency, indicating the effective removal of fine particles and organic matter residues after the sorption stage. Such high filtrate quality confirms the validity of using membrane filtration as the final stage of treatment, especially in the case of complex wastewater, such as that from the coffee industry. In addition, the results obtained indicate the potential for using such treated water in disinfection processes (ozonation, UV radiation), which in the long term enables its reuse.

A similar trend was observed in earlier studies using pure caffeine as a model contaminant [23]. At that time, the degree of TOC removal using a 0.2 µm CN membrane exceeded 90%, while that of phosphates exceeded 80%. In the current study, the efficiency was slightly lower, especially for phosphates, which can be explained by the greater complexity of the model wastewater composition. The polyphenols, fats, and aromatic compounds present in the coffee bean extract may have contributed to the intensification of membrane fouling, limiting their filtration efficiency. Hence, the integration of sorption processes with advanced separation methods, such as membrane filtration, is particularly effective in treating wastewater with a complex composition. As shown by Hernández Gómez et al. (2021) [41], the use of membrane processes such as ultrafiltration and reverse osmosis enables effective water recovery from food industry wastewater, achieving over 95% treatment efficiency. The authors emphasize that the efficiency of membranes increases significantly when preceded by a pre-treatment stage, e.g., sorption or sedimentation, which reduces fouling. Similar conclusions were presented in the research by Pervez et al. (2021) [42], pointing to the effectiveness of membrane technologies in the sustainable treatment of food industry wastewater, especially in the removal of suspended solids, fats, salts, and organic compounds. The research by Raza et al. (2019) [43] confirmed the high efficiency of membrane processes in eliminating phenolic compounds from industrial wastewater, which indicates their usefulness in the final treatment of wastewater from the coffee industry. In turn, the studies by Obotey Ezugbe et al. (2020) [44] and Yang et al. (2011) [45] emphasize that combining membrane filtration with biological or sorption processes enables more effective removal of poorly biodegradable pollutants, including pharmaceuticals and detergents, significantly improving the quality of the filtrate obtained. In addition, sorption plays an important protective role—it reduces the amount of organic and colloidal substances reaching the membrane, limiting fouling, extending its service life, and reducing operating costs [46]. Research by Zhang et al. (2024) [47] confirms that combining membrane filtration with other processes, such as photocatalytic processes, further increases the effectiveness of pollutant removal and reduces fouling, enabling continuous and sustainable wastewater treatment. In the context of the research conducted, the membrane filtration stage following the sorption process thus performs a similar treatment function, enabling the effective removal of remaining organic compounds and obtaining high-quality filtrate ready for further treatment processes or reuse.

### 4.4. Multi-Stage Wastewater Treatment with Disinfection

Advanced oxidation processes (AOPs), such as ozonation and UV radiation, are among the most effective methods for removing organic pollutants from wastewater, converting them into less toxic compounds. Ozonation generates hydroxyl radicals with strong oxidizing potential, which effectively break down poorly biodegradable substances, including phenols and pharmaceutical compounds. The literature emphasizes that the combination of ozonation and UV radiation has a synergistic effect, significantly increasing the effectiveness of pollutant removal compared to the use of single processes [48].

In our own research, the final stage of the treatment process was wastewater disinfection, which aimed to remove the microbiological load and improve biological safety. After membrane filtration, the samples were subjected to ozonation and then exposed to UV radiation. The combination of processes used made it possible to effectively reduce the number of microorganisms, including pathogenic bacteria, and improve physicochemical parameters such as color and clarity. Ozone also proved to be highly effective in oxidizing organic compounds, while UV radiation was a complementary process, ensuring more complete disinfection and minimizing the risk of survival of microorganisms resistant to a single treatment. The results obtained confirm that the combination of ozonation and UV radiation is an effective strategy for final wastewater treatment in hybrid systems.

The well-known UV/O_3_ process leads to the intensive production of hydroxyl radicals, which increases the effectiveness of disinfection and degradation of organic pollutants. In an aquatic environment, ozone reacts with hydroxyl and peroxyhydroxyl anions, generating strong oxidants responsible for the rapid decomposition of organic substances. The UV/O_3_ system has been shown to effectively remove TOC and color from wastewater, although the complete mineralization of phenolic compounds may be limited [49]. Research by Chen et al. (2007) [50] confirms that the appropriate selection of ozone dose and UV radiation intensity allows for the effective removal of TOC and toxic compounds such as dinitrotoluene and trinitrotoluene. The high effectiveness of ozonation has also been confirmed in the removal of compounds responsible for wastewater color, especially polyphenolic compounds. Similarly, research by Andreozzi et al. (2008) [51] confirmed that ozonation of wastewater from olive mills for 120 min allowed for the removal of polyphenols and COD by 82.4% and 59.8%, respectively. Similar results were obtained by Sangave et al. (2007) [52] in their studies, achieving 79% COD removal in distillery wastewater and 90% color removal in molasses fermentation wastewater. The combination of ozonation with photocatalysis or UV radiation significantly increases the efficiency of the process, shortening the reaction time and enabling the simultaneous removal of COD, BOD, and organic substances in various types of wastewater [34]. Studies by Jorge et al. (2021) [53] indicate that the combination of coagulation–flocculation–decantation (CFD) processes with ozonation significantly improves the removal of pollutants in wine wastewater, reducing the content of polyphenols and organic substances and lowering their toxicity. Additionally, the integration of ozonation with UV-C radiation and Fe^2+^ ions allows for further removal of total organic carbon (TOC), achieving an efficiency of up to 66%. A key factor determining the effectiveness of the process is the appropriate selection of operating parameters, such as pH and Fe^2+^ concentration, which determine the optimal conditions for the oxidation reaction. The integration of CFD, ozonation, and UV processes has a strong synergistic effect, leading to shorter reaction times and increased disinfection and contaminant removal efficiency.

In recent years, the literature on the treatment of agricultural and food wastewater has clearly shown a trend toward the development of integrated treatment systems that combine biological processes, sorption, membrane filtration, and advanced oxidation processes. Such approaches are part of the concept of next-generation treatment plants, focused on water recovery and the removal of persistent pollutants and micropollutants [54]. For example, systems based on membrane bioreactors (MBR) combined with further treatment processes, such as nanofiltration or reverse osmosis, achieve very high removal efficiencies for organic compounds, often exceeding 90% for COD, total nitrogen (>75%), and total phosphorus (>74%), and also ensure effective pathogen removal, enabling water reuse. It is also noted that integrating MBR with oxidation processes (e.g., ozonation) or adsorption can further improve effluent quality and reduce the load on the biological treatment system [55].

These results are consistent with the observations obtained in this study, confirming the high efficiency of integrated, multi-stage wastewater treatment systems combining biological processes, sorption, membrane filtration, and advanced oxidation (ozonation and UV radiation). This comprehensive approach enables the effective removal of poorly biodegradable compounds, the elimination of microbiological load, and significant water recovery, which creates the possibility of its reuse. The integration of biological and physicochemical processes is therefore a promising strategy for increasing the efficiency of wastewater treatment and rational water management in the coffee industry. The research conducted is an important preliminary step, setting the direction for further work on the optimization of integrated wastewater treatment technologies. The results obtained allow the identification of key factors influencing the efficiency of individual stages of the process and indicate areas requiring further analysis in the future. Further research should focus on refining the operating parameters, economic analysis, and assessment of the environmental benefits of the proposed solution.

### 4.5. Energy Consumption and Cost Analysis of the Integrated Treatment System

An important aspect of evaluating the proposed multi-stage technological system is the analysis of its energy demand and operational costs, which largely determine the feasibility of its implementation at a technical scale. According to the literature, the energy consumption of advanced wastewater treatment systems strongly depends on process configuration and may vary widely; however, systems incorporating advanced treatment stages such as membrane filtration and adsorption are typically reported to operate within the range of approximately 0.5–2.0 kWh/m^3^ [56].

In conventional biological treatment systems, such as activated sludge processes, the unit energy consumption typically ranges from 0.30 to 0.65 kWh/m^3^ [57]. The main contributor to energy demand is aeration, which may account for 45–75% of the total energy consumption of wastewater treatment plants [58]. Therefore, in the case of the SBR reactor applied in this study, the energy demand can be considered consistent with values reported for activated sludge systems, with aeration and mixing being the dominant energy-consuming processes.

Another important component is adsorption on activated carbon. This process is generally characterized by relatively low direct energy demand compared to pressure-driven processes; however, its overall cost is strongly influenced by sorbent production, replacement, and regeneration requirements. The literature emphasizes that adsorption is often economically justified only when integrated with other processes, especially in hybrid treatment systems combining adsorption and advanced oxidation [56]. In this context, the selection of the dynamic sorption variant in the present study can be considered advantageous not only in terms of treatment efficiency but also from an operational perspective, as flow-through systems are more compatible with continuous treatment configurations.

Membrane filtration, despite its high separation efficiency, is among the most energy-intensive stages of advanced treatment systems. The inclusion of membrane processes in water reuse systems significantly increases total energy consumption, often exceeding that of conventional biological treatment [56]. Moreover, membrane fouling leads to increased hydraulic resistance, resulting in higher energy demand and additional operational costs associated with cleaning and membrane replacement. The integration of sorption as a pre-treatment step, as applied in this study, may effectively reduce fouling and improve the overall economic performance of the system.

The final disinfection stage, based on ozonation and UV radiation, is also associated with high energy demand. Ozone generation is an energy-intensive process, requiring on the order of 10–20 kWh per kg of O_3_ produced, depending on the technology used [59]. In addition to energy consumption, ozonation contributes significantly to operational costs due to electricity demand and oxygen supply requirements. The inclusion of UV radiation further increases total energy consumption, although the combined O_3_/UV process provides high efficiency in the degradation of refractory organic compounds and ensures effective disinfection.

From an economic perspective, the total cost of advanced wastewater treatment systems depends on multiple factors, including energy prices, system scale, and process complexity. Operational costs are typically dominated by energy consumption, sludge management, and chemical usage [60]. Although advanced hybrid systems (e.g., combining membrane filtration and ozonation) are associated with higher costs compared to conventional treatment, they enable significantly improved effluent quality and water reuse potential, which may justify their implementation under strict environmental regulations.

In summary, the multi-stage technological system proposed in this study should be considered highly effective but relatively energy- and cost-intensive. The highest energy demand is associated with aeration, membrane filtration, and ozonation processes. At the same time, the integration of individual treatment stages (biological, sorption, and membrane) enables partial cost reduction through synergistic effects, such as decreased membrane fouling and improved overall process efficiency. Future research should focus on optimizing operational parameters, developing low-cost sorbents, and improving the energy efficiency of advanced oxidation processes to enhance the overall feasibility of such integrated systems.

## 5. Conclusions

The presented research contributes new knowledge to the issue of wastewater treatment arising from the processing of roasted coffee beans, which until now has been analyzed much less frequently than wastewater from the wet processing of green beans. The use of model wastewater with the addition of roasted coffee extract made it possible to reproduce the composition of pollutants characteristic of the extraction and washing processes of technological installations in the production of instant coffee, which enabled the evaluation of the effectiveness of an advanced, integrated treatment system. The results obtained confirm that the combination of biological processes, activated carbon sorption, membrane filtration, and two-stage disinfection (ozonation and UV radiation) is an effective solution for removing poorly biodegradable organic compounds present in this type of wastewater. At the same time, the use of a multi-stage technological system significantly increases the degree of wastewater treatment and creates real opportunities for water recovery with parameters that allow its reuse in industrial processes. The results of the research indicate high potential for further development of integrated wastewater treatment technologies for the coffee industry, which may contribute to reducing the environmental pressure of this dynamically developing sector and support the implementation of circular economy principles.

Based on the research results and literature review, the following conclusions were drawn:

### 5.1. Research Substrate

1. There is still a lack of sufficient research in scientific literature on the treatment of wastewater from the coffee industry using integrated technological systems involving biological processes, sorption, membrane filtration, and disinfection.
2. The studies conducted have shown that the specific composition of wastewater containing coffee bean extract significantly affects the efficiency of individual treatment stages, especially with poorly biodegradable organic compounds.
3. Wastewater with a high concentration of coffee bean extract proved difficult to treat effectively and to achieve the parameters required by applicable environmental standards.

### 5.2. Biological Treatment

1. An analysis of two variants of sequential biological reactor operation phases showed that better results were obtained in variant II, which used an alternating aeration and mixing system, as opposed to variant I, which was based on 22 h of continuous mixing and aeration of the reactors.
2. In the selected variant of reactor operating phases, the following degrees of pollutant removal were achieved: total organic carbon—69.3–88.5%, ammonium nitrogen—0–65.2%, phosphates—25.4–85.9%, turbidity—33.7–90.5%. At the same time, an increase in pH values was observed, from 5.64–6.77 before the process to 5.09–7.45 after its completion.
3. Biological wastewater treatment enabled significant removal of pollutants. Still, its effectiveness was insufficient due to the complex composition of the model wastewater, which was enriched with coffee bean extract in three different concentrations. Therefore, it was necessary to apply further treatment stages, including sorption, membrane filtration, and disinfection using ozone and UV radiation.

### 5.3. Integration of Biological Wastewater Treatment with Activated Carbon Sorption Process

1. Analysis of sorption under static and dynamic conditions showed higher efficiency and shorter contact time in the dynamic process, in which the sample flows through the activated carbon bed.
2. The dynamic method ensured further removal of turbidity, total organic carbon, and phosphates, as well as greater operational and economic efficiency, and was therefore selected for further research.
3. Slightly better results were obtained using WG-12 activated carbon.
4. Under dynamic conditions, WG-12 activated carbon allowed for further effective removal of total organic carbon (0–56.8%), phosphates (0–24.6%), and turbidity (0–84.9%), while increasing the pH value of the samples (8.85–9.09) and preparing the sample for the next stage—membrane filtration.

### 5.4. Integration of Biological Wastewater Treatment with Sorption and Membrane Filtration Processes

1. After completion of the sorption process, the samples were subjected to membrane filtration, with the best results achieved using cellulose nitrate (CN) membranes with a pore diameter of 0.2 µm, which were used in the next stage of the tests.
2. Membrane filtration also allowed for further removal of turbidity (88.1–98.1%) and maintenance of the pH value within the optimal range of 5.11–7.55.

### 5.5. Multi-Stage Wastewater Treatment with Disinfection

1. The integrated, multi-stage technological system used allowed for the effective combined removal of total organic carbon (82.4–95.4%), ammonium nitrogen (0–77.4%), phosphates (0–39.9%), and turbidity (96.3–99.8%), while stabilizing the pH value (4.02–7.25). The variability in ammonium nitrogen and phosphate removal was mainly due to transformations during biological treatment (ammonification and phosphorus release), differences in SBR operation, and limited effectiveness of subsequent stages for dissolved nutrients.
2. In addition, the use of two-stage wastewater disinfection (ozonation and UV radiation) enabled the effective removal of microorganisms and further improvement of physicochemical parameters, including the color of treated wastewater.

## Figures and Tables

**Table 1:** General characteristics of the freshly prepared research substrate (own elaboration).

Indicator	Unit	Synthetic Wastewater with Coffee Extract			Synthetic Wastewater
		1 g/L	10 g/L	20 g/L	
Ph	-	6.77	5.76	5.64	6.43
Turbidity	NTU	11.8	64.2	160.0	8.62
Phosphates	mg P-PO_4_/L	35.77	19.81	27.93	2.68
Ammonium nitrogen	mg N-NH_4_/L	5.50	6.6	9.3	6.0
Total organic carbon	mg O_2_/L	212.1	922.2	1750.0	82.64

**Table 2:** The specified reactor operating phases for variants I and II (own elaboration).

Names of the Individual Phases for Variant I of the Reactor Operating Cycle	Duration of Each Phase of the Cycle	Names of the Individual Phases for Variant II of the Reactor Operating Cycle	Duration of Each Phase of the Cycle
Sedimentation and decantation	1.5 h	Sedimentation and decantation	1.5 h
Exchange	0.5 h	Exchange	0.5 h
Mixing and aeration	22 h	Mixing	1 h
		Aeration	8 h
		Mixing	4 h
		Aeration	7 h
		Mixing	2 h

**Table 3:** Parameters of the activated carbons used in the research [26,27].

	WACC	WG-12
Specific surface area, minimum	1000 m^2^/g	800–1100 m^2^/g
Iodine value, minimum	1000 mg/g	1000 mg/g
Dechlorination capacity	<5 cm	
Humidity, maximum	5%	2%
Ash content, maximum	5%	15%
pH	~8	~7
Hardness, minimum	98%	95%
Bulk density	480 ± 30 g/L	450 ± 30 g/L
Grain size	0.6–2.4 mm	1.0–1.5 mm

## Data Availability

The original contributions presented in this study are included in the article. Further inquiries can be directed to the corresponding author.

## Abbreviations

The following abbreviations are used in this manuscript:

AOPs	Advanced Oxidation Processes
BOD	Biochemical Oxygen Demand
CA	Cellulose Acetate
CFD	Coagulation–Flocculation–Decantation process
CN	Cellulose Nitrate
CN-CA	Mixed cellulose esters
COD	Chemical Oxygen Demand
HRT	Hydraulic Retention Time
ICO	International Coffee Organization
MBR	Membrane Bioreactor
SBR	Sequencing Batch Reactors
SWW	Synthetic Wastewater
TOC	Total Organic Carbon
